# Biologically synthesis of gold nanoparticles using *Cirsium japonicum* var. *maackii* extract and the study of anti-cancer properties on AGS gastric cancer cells

**DOI:** 10.7150/ijbs.77734

**Published:** 2022-09-21

**Authors:** Xiao-jie Mi, Hye-Ryung Park, Sanjeevram Dhandapani, Sanghyun Lee, Yeon-Ju Kim

**Affiliations:** 1 Graduate School of Biotechnology, and College of Life Science, Kyung Hee University, Yongin-si, 17104, Gyeonggi-do, Republic of Korea.; 2 Department of Plant Science and Technology, Chung-Ang University, Anseong 17546, Republic of Korea.

**Keywords:** *Cirsium japonicum*, Gold nanoparticles, Gastric cancer, Ferroptosis

## Abstract

Plant extract-mediated synthesis of metal nanoparticles (NPs) is an eco-friendly and cost-effective biosynthesis method that is more suitable for biological applications than chemical ones. We prepared novel gold NPs (AuNPs), *Cirsium japonicum* mediated-AuNPs (CJ-AuNPs), using a biosynthetic process involving *Cirsium japonicum* (Herba Cirsii, CJ) ethanol extract. The physicochemical properties of CJ-AuNPs were characterized using spectrometric and microscopic analyses. The *in vitro* stability of CJ-AuNPs was studied for 3 months. Moreover, the selective human gastric adenocarcinoma (AGS) cell killing ability of CJ-AuNPs was verified in cancer and normal cells. An *in vitro* study revealed that CJ-AuNPs trigger oxidative stress and iron-dependent ferroptosis in AGS cells. Mechanistically, CJ-AuNPs induced mitochondrial reactive oxygen species (ROS), Fe^2+^, and lipid peroxidation accumulation, and mitochondrial damage by destroying the glutathione peroxidase-4 (GPX4)-dependent antioxidant capacity. Furthermore, in a xenograft mouse model implanted with AGS cells, treatment with 2.5, 5, and 10 mg/kg CJ-AuNPs for 16 days reduced tumor xenograft growth in a dose dependent manner *in vivo* without systemic toxicity. These results demonstrate that CJ-AuNPs exert anticancer effects *in vitro* and *in vivo* by inducing ferroptosis-mediated cancer cell death. This study, based on green-synthesized nanodrug-induced ferroptosis, provides new insight into potential developments in cancer therapies.

## Introduction

Gastric cancer (GC), the fourth most common cancer globally, has the second-highest mortality rate among all cancers 1. Although treatable by chemotherapy, surgical interventions, or a combination of these, remission is often associated with resistance, poor prognosis and disease relapse 2. Hence, identifying potential anticancer candidates for GC is strongly recommended. Recently, nanobiotechnology has attracted attention as an alternative cancer treatment approach.

Over the last few decades, the field of nanobiotechnology has developed rapidly, incorporating knowledge and research in materials science, chemistry, and biology 3. Nanoparticles (NPs) occupy a pivotal position in various fields, such as biomedical development, pharmaceutical research, diagnosis, and drug delivery 4. In particular, nanotechnology has great potential in the early stages of cancer detection and treatment because of its shape and size, which contribute to its targeted and toxic potential 5. Gold nanoparticles (AuNPs) are among the most widely used nanomaterials in biomedical therapeutics because of their strong surface plasmon resonance and highly optimized protocols 6. However, AuNPs synthesis by conventional physical and chemical methods often employs the hazardous reducing and stabilizing agents, which can remain in the end product and threaten human health 7. Thus, plant-based synthesis of AuNPs emerges as a safer option since phytomedicine has been used for decades in traditional medicine 8. Various phytochemicals extracted from plants have been used for treating diseases such as cancer. These phytochemicals therein play an essential role in the bio-reduction of Au^3+^ to produce their respective stable AuNPs without toxic ligands. Subsequently, they form a surface coating on AuNPs, rendering AuNPs bioactive for medical application 7. A large number of groups have been engaged in the green synthesis of biocompatible AuNPs with different size and shape, taking the bioactive compounds from plants 9. It has been demonstrated that polyphenols from plants can be used as reducing agents for the development of new AuNPs-based anti-cancer agents. A recent study showed that the AuNPs functionalized with phytochemicals from Compositae plant induced tumor destruction on an experimental model of skin cancer in murine, without causing the necrosis of healthy tissue 10.

*Cirsium japonicum* var. *maackii* (Herba Cirsii, Compositae) is a perennial herbaceous species and a well-known traditional herbal preparation in China, Japan, and Korea 11. It has a long history as a medicinal herb used to treat various psychophysiological diseases, including chronic hepatitis and Weil's disease 12,13. Recently, the therapeutic benefits of *Cirsium japonicum* (CJ) have been reported to include antidiabetic 14, anti-tumor 15, and anti-inflammatory effects 16. In addition, CJ ethanol extracts have notable antioxidant and free radical scavenging abilities owing to the presence of flavonoids, flavonolignans, sterols, volatile oils, long-chain alcohols, and other polyphenolic compounds 17. The high flavonoid and polyphenolic content of CJ make it a valuable source of bioactive compounds capable of reducing metal ions and acting as a capping agents in the green synthesis of AuNPs 18,19. However, the potential of CJ to mediate AuNPs biosynthesis has currently not been investigated.

This study aimed to develop and characterize a new green technique to synthesize the AuNPs using CJ ethanol extract (CJ-AuNPs). The synthesized AuNPs were screened for their biological effects on cervical cancer (Hela), gastric cancer (AGS), colon cancer (HT-29), and liver cancer (HepG2) cell lines, and the findings were compared with the effects of CJ ethanol extract alone. Moreover, we explored the mechanisms underlying the anti-tumor activities of CJ-AuNPs *in vivo* and *in vitro*, particularly their involvement in ferroptosis. Recent reports suggest that nanoparticles-induced ferroptosis offers the opportunity to inhibit cancer cells development, which can aid clinicians in overcoming challenges associated with conventional tumor therapies that focus on inducing apoptosis. Although several small-molecule inducers have been confirmed to trigger ferroptosis in cancer cells, their rapid renal clearance and systemic toxicity limit their clinical applicability^20^. Therefore, nano-inducers that activate ferroptotic cell death are highly desirable in cancer therapies. This study provides basic data for the development of green synthesized CJ-AuNPs as a ferroptosis inducer in cancer cells. To the best of our knowledge, this is the first study to report the anticancer properties of CJ-mediated AuNPs.

## Materials and methods

### Chemicals and Reagents

Dulbecco's modified Eagle's medium (DMEM), Roswell Park Memorial Institute (RPMI)-1640 medium (RPMI), penicillin-streptomycin, and fetal bovine serum (FBS) were purchased from GenDEPOT (Katy, TX, USA). Dimethyl sulfoxide (DMSO) and 3-(4,5-Dimethylthiazol-2-yl)-2,5-diphenyltetrazolium bromide (MTT) were obtained from Sigma-Aldrich (St. Louis, MO, USA). The Live/dead cell viability/cytotoxicity kit was provided from Thermo Fisher Scientific (Cambridge, MA). Hydrogen tetrachloroaurate hydrate (HAuCl_4_•3H_2_O) was supplied from Strem Chemicals, Inc. (Newburyport, MA, USA).

### Preparation of plant materials

The dried aerial part of CJ was purchased commercially from Imsil Herbal Medicine (Imsil-gun, Jeollabuk-do, Korea) and extracted with 10 times the volume of 70% ethanol. The mixture was refluxed at 65 °C for 3 h. The filtrate was collected after vacuum filtration at 25 °C, and then evaporated with a rotary evaporator (RE-52A, Shanghai yarong biochemical instrument factory) under reduced pressure at 45 °C. The total CJ ethanol extract was yielded as the viscous residue. After being dried in a dry oven (HK-DO135F, Hankook machine, Korea) at 40 °C, the obtained CJ ethanol extract was stored at 4 °C for further use.

### Preparation and optimization of CJ-AuNPs

To prepare CJ-AuNPs, the optimized conditions were monitored in the previous study^21^. CJ ethanol extract was dissolved in distilled water and passed through a 0.22 µm filter (Advantec) before use. The filtered CJ was mixed with HAuCl_4_·3H_2_O solution at different concentrations. The reaction mixture was incubated in a shaking incubator (MSH-20A; Daihan Scientific, Gangwon-do, Korea). The optimized conditions for the CJ-AuNPs biosynthesis were explored in terms of concentrations of CJ ethanol extract and HAuCl_4_·3H_2_O, incubation time, and reaction pH value. The completion of biosynthesis was decided by visualization of changed color (from yellow to purple), following determination of optical density using UV-visible (Vis) spectrophotometer (Cary 60; Agilent Technologies, Santa Clara, CA, USA). To remove the soluble materials, the mixture was centrifuged (12,000 rpm, 20 min) using a centrifuge (Smart R17 Plus, Hanil Scientific Inc., Korea), and the precipitated particles were washed with distilled water. This process was repeated four more times to purify the particles. Finally, the CJ-AuNPs were stored at 4 °C for further cell treatment.

### Physicochemical characterization of the CJ-AuNPs

Ultraviolet-visible (UV-Vis) spectrophotometer (Cary 60) was used to confirm the maximal absorbance of CJ-AuNPs at the range of 300-800 nm. Field-emission-transmission electron microscopy (FE-TEM; JEM-2100F, JEOL, Ltd., Tokyo, Japan) coupled with selected area electron diffraction (SAED) and energy-dispersive X-ray spectrometry (EDX) was used at a voltage of 200 kV to measure the particle size, microscopic morphology, elemental composition, and crystalline nature of the CJ-AuNPs. Briefly, the purified CJ-AuNPs were dispersed in water and dripped on carbon-coated copper grid, and subsequently dried at room temperature before transferring to the TEM.

The field emission scanning electron microscope (FE-SEM; 7800F, JEOL, Tokyo, Japan) was used to determine the morphology, purity, structure, and elemental distribution of CJ-AuNPs. Furthermore, the hydrodynamic size and size distribution of CJ-AuNPs were measured in water using a dynamic light scattering (DLS) particle size analyzer (Otsuka Electronics, Shiga, Japan). Powder X-ray diffraction (XRD) patterns were recorded to characterize the crystal structures of the CJ-AuNPs. To identify the functional groups on the surface of the CJ-AuNPs, fourier-transform infrared (FT-IR) spectrometer (Spectrum One FTIR Spectrometer; PerkinElmer, Waltham, MA, USA) was analyzed in the range 400-4000 cm^-1^.

### Statistical analysis

All data are expressed as means ± standard error (SEM). Statistical analysis was performed using SPSS 17.0 Statistics (SPSS, Inc., Chicago, IL, USA). Statistically significant between the groups were analyzed using one-way analysis of variance (ANOVA) followed by Dunnett's test. *p*<0.05 were considered statistically significant.

For further details regarding the materials and methods used, please refer to the Supplementary Information 1.

## Results and Discussion

### Chemical composition of CJ ethanol extract

**Figure 1:**
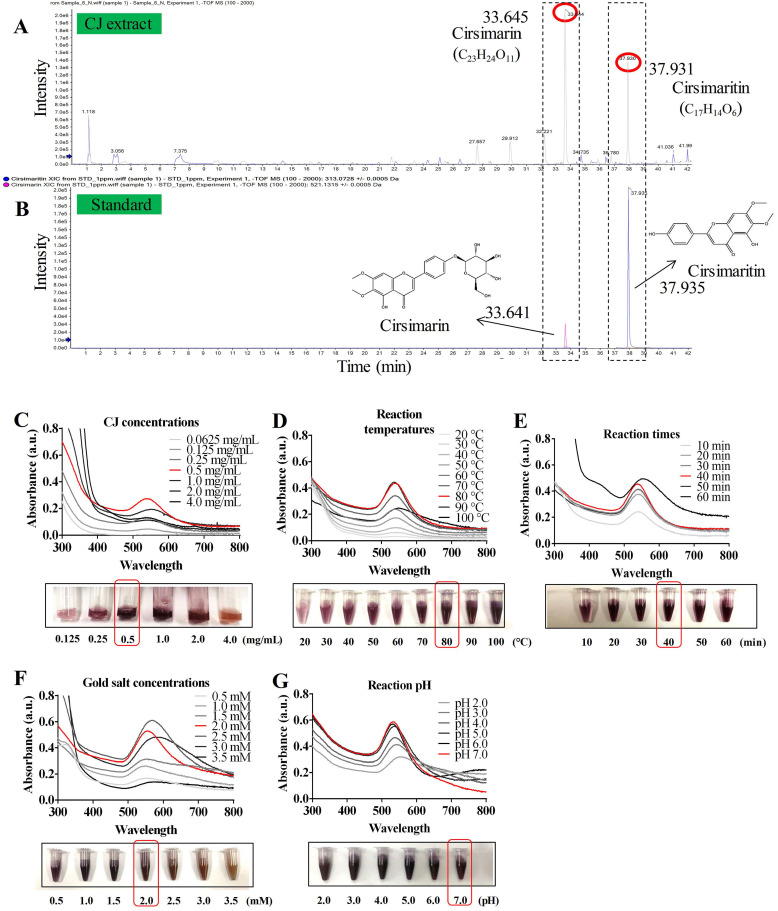
** Synthesis and optimization of CJ-AuNPs.** LC-MS analysis present the main chemical compositions are cirsimarin and cirsimaritin in CJ ethanol extract **(A)**; HPLC method to determine and quantify cirsimarin and cirsimaritin in CJ-AuNPs **(B)**; Optimization of reaction condition, including reaction CJ concentrations **(C)**, reaction temperatures **(D)**, reaction times **(E)**, gold salt concentrations **(F)**, and reaction pH **(G)** for the large-scale bioreduction of CJ-AuNPs.

Several studies have reported the presence of various phytochemicals, including flavonoids, flavones, sterols, long-chain alcohols, and other polyphenolic compounds, in CJ ethanol extract 22. The significant antioxidant properties of these phytochemicals offer the possibility of reducing metallic ions in the green synthesis of AuNPs 18,19. In this study, the main compounds in CJ ethanol extracts were identified using LC-MS. As a result, 126 common compounds were detected in the CJ ethanol extract by the total negative/positive ion mode (Table S1), and cirsimarin was observed at its highest peak in the CJ ethanol extract at a retention time of 33.645 min (Fig. 1A). The second major compound was identified as cirsimaritin, with a retention time of 37.931 min (Fig. 1A). Cirsimarin and cirsimaritin were used as standards to confirm their contents in the CJ ethanol extract. As shown in Fig. 1B, the contents of cirsimarin and cirsimaritin were 98.83 µg/mg and 5.80 µg/mg in CJ ethanol extract, respectively. Cirsimarin and cirsimaritin are flavonoids and flavones, respectively. Plant flavonoids and flavones possess various functional groups capable of reducing metal ions to NPs; therefore, they can act as reducing and capping agents in AuNPs production 18. Lee *et al*. successfully prepared AuNPs using the flavonoid quercetin as a reductant using a green approach for potential nano architechtonic applications 23. These results suggest that the CJ ethanol extract may have the potential to stabilize and reduce Au^3+^ to Au^0^ to obtain AuNPs with improved biological properties. However, to the best of our knowledge, CJ-mediated green synthesis of AuNPs has not yet been reported. This study aimed to biosynthesize CJ-AuNPs following a one-step reaction in which the biological molecules of CJ are utilized as reducing and stabilizing agents.

### Optimized condition for biosynthesis of CJ-AuNPs

The AuNPs formation process is affected by various synthesis parameters. Therefore, an ultraviolet-visible (UV-Vis) spectrophotometer was used to optimize the synthesis parameters of CJ-AuNPs 24. As shown in Fig. 1C-G. The reaction CJ concentrations (Fig. 1C), reaction temperatures (Fig. 1D), reaction times (Fig. 1E), HAuCl_4_·3H_2_O (gold salt) concentrations (Fig. 1F), and reaction pH (Fig. 1G) were optimized for the large-scale bioreduction of CJ-AuNPs. Optimal conditions were established by visualizing the color change from yellow to dark purple and screening the absorbance from 300 nm to 800 nm. First, the UV-Vis spectra of CJ-AuNPs synthesized with different concentrations of CJ extract were recorded. As shown in Fig. 1C, when different concentrations (0.0625, 0.125, 0.25, 0.5, 1.0, 2.0, and 4.0 mg/mL) of CJ and 1 mM HAuCl_4_•3H_2_O solution at pH 3.0 were reacted at 60 °C for 30 min, surface plasmon wavelengths of AuNPs were observed at from 300 nm to 800 nm, the large-scale bioreduction of AuNPs was observed when the concentration of CJ was 0.5 mg/mL. Therefore, 0.5 mg/mL CJ was selected to further optimize the reaction temperature. When 0.5 mg/mL CJ and 1 mM HAuCl_4_•3H_2_O solution were reacted at different temperatures for 30 min, absorption intensity was improved by the increasing temperature range from 20 °C to 80 °C (Fig. 1D). The large-scale bioreduction of CJ-AuNPs was observed at 80 °C and 90 °C (Fig. 1D). However, the absorption intensity was decreased after heating at 100 °C, which could mainly be due to the degradation of plant metabolites and aggregation of NPs at high temperatures 25. Fig. 1E illustrates the effect of reaction times on the formation of CJ-AuNPs. The major absorption peaks of CJ-AuNPs were more significant when the reaction time was 40 min. Furthermore, when HAuCl_4_•3H_2_O concentration was 2.0 mM, major absorption peaks at 545 nm were more significant. Moreover, the steepest peak for the CJ-AuNPs was observed at a high pH (7.0) (Fig. 1G). This may indicate that the chemical reduction rate of gold ions is directly proportional to the pH value to some extent, which may be due to the greater availability of free OH groups under relatively high pH conditions 26.

**Figure 2:**
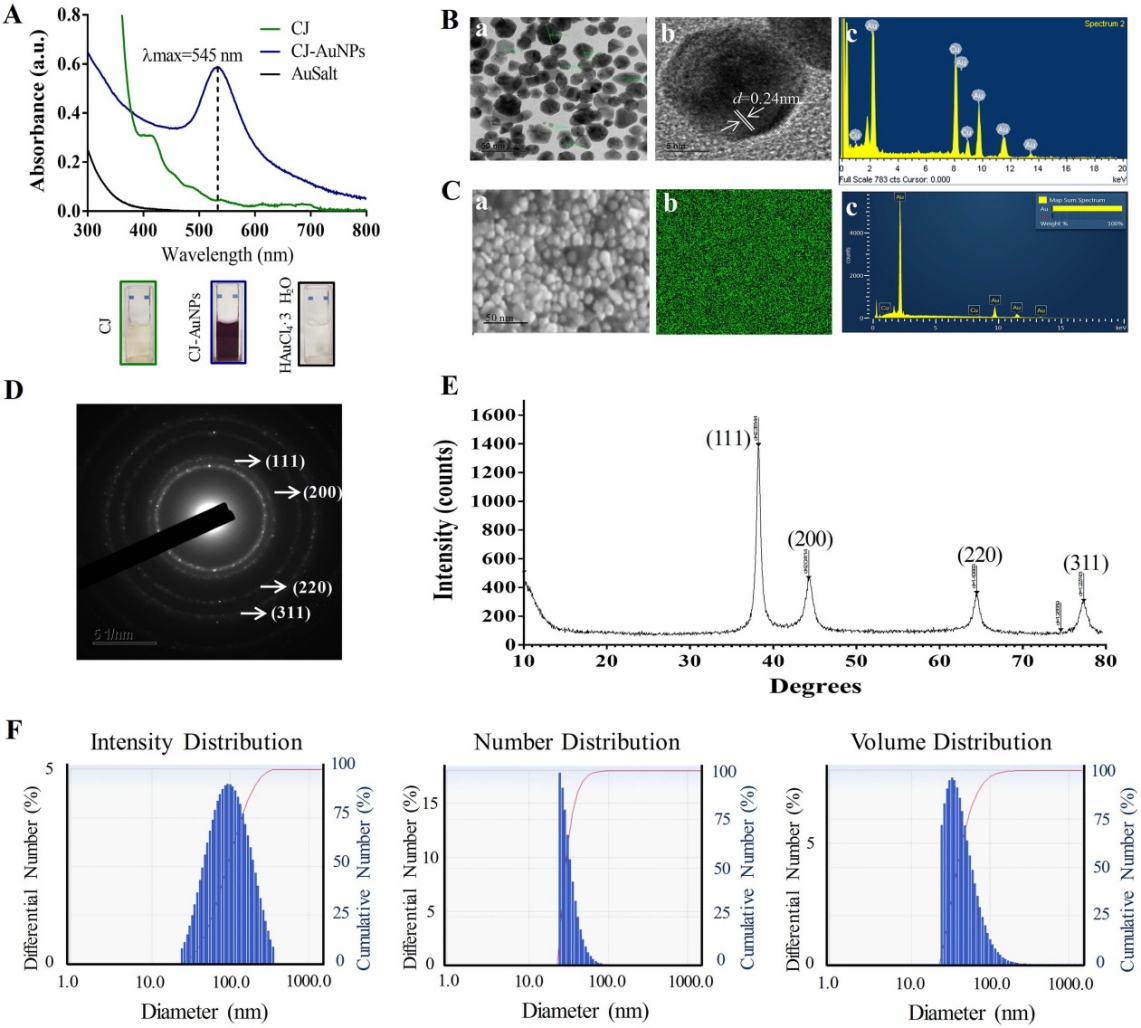
Characterization of CJ-AuNPs. UV-Vis absorption spectroscopy analysis of CJ, CJ-AuNPs, and gold salt **(A)**; TEM micrograph of CJ-AuNPs in which the scale bar represents 50 nm (a) and 5 nm (b); Energy-dispersive X-ray spectroscopy (EDX, c) analysis **(B)**; FE-SEM pattern (a) and gold distribution (b) of CJ-AuNPs with a EDX analysis (c) **(C)**; Selected area electron diffraction (SAED) pattern of CJ-AuNPs **(D)**; The X-ray diffraction (XRD) pattern (right) of CJ-AuNPs **(E)**; Size distribution of the CJ-AuNPs using dynamic light scattering (DLS) with respect to intensity, number, and volume **(F)**.

The UV-Vis absorption spectra of 0.5 mg/mL CJ extract, 2 mM HAuCl_4_•3H_2_O, and optimum CJ-AuNPs were then assessed and compared. As shown in Fig. 2A, no specific absorption peak was observed on both CJ and gold salt from 300 to 800 nm, CJ-AuNPs exhibited a robust peak at 545 nm. The visible color difference was observed between CJ (light yellow) and CJ-AuNPs (deep purple) during the NP synthesis process. These results exhibited that when 0.5 mg/mL CJ and 2.0 mM HAuCl_4_·3H_2_O in the solution at pH 7.0 were reacted at 80 °C for 40 min, CJ-AuNPs synthesis yielded the best production outcomes. These data demonstrate that the CJ-AuNPs were synthesized under optimal conditions and used for subsequent experiments. To our knowledge, this is the first study to investigate the possibility of applying nanoformulations using CJ extract.

### Physicochemical characterization of CJ-AuNPs

The structural properties (size and morphology) of synthesized CJ-AuNPs were determined using three independent techniques, including field emission-transmission electron microscopy (FE-TEM), field emission-scanning electron microscopy (FE-SEM), and dynamic light scattering (DLS). FE-TEM analysis was performed to identify the predominant shape, size, and crystalline structure of CJ-AuNPs 27. As shown in Fig. 2B(a-b), TEM measurements of CJ-AuNPs revealed the majority of spherical or polygonal-shaped surface morphologies with lattice fringes with sizes varying from 6 to 38 nm. The energy dispersive X-ray (EDX) spectrum demonstrated the highest characteristic peak of metallic gold at 2.2 keV, suggesting that gold was the predominant element in the CJ-AuNPs (Fig. 2B(c)). FE-SEM was employed to determine the metallic core of the NPs. As shown in Fig. 2C(a), the compact spherical morphologies with high aggregation were observed, suggesting high surface energy of the CJ-AuNPs. The purity of the CJ-AuNPs was determined by elemental mapping and EDX spectroscopy. As shown in Fig. 2C(b), the distribution of elemental gold (green color) was clearly discernible within the synthesized CJ-AuNPs, suggesting successful polymerization between gold ions and CJ. This is also consistent with the results of EDX spectroscopy (Fig. 2C(c)), in which only gold element peaks were observed in the CJ-AuNPs, indicating that the CJ-AuNPs were successfully synthesized with high purity. To confirm the diffraction pattern of multiple particles and crystalline nature of the CJ-AuNPs, crystallographic techniques, such as selected area electron diffraction (SAED) and X-ray diffraction (XRD) were used. As shown in Fig. 2D, the SAED pattern revealed four ring features (111, 200, 220, and 311) in the lattice planes, confirming the crystalline structure of the CJ-AuNPs. This observation was verified by the XRD results, which included diffraction peaks at 2θ values of 38.12°, 44.53°, 64.68° and 77.93°, corresponding to four characteristic peaks of gold at the (111), (200), (220), and (311) lattice planes of Bragg's reflection (Fig. 2E) 28.

The hydrodynamic size (Z-average) of the CJ-AuNPs was determined using DLS, including the intensity, number and volume distribution. As shown in Fig. 2F, the polydispersity of CJ-AuNPs was apparent from the wide intensity distribution, which was consistent with previously reported FE-TEM and FE-SEM results. The hydrodynamic diameter of CJ-AuNPs was substantially changed due to the CJ phytochemical coating, which was confirmed from the DLS measurement yielding an average of 220.8 nm with a moderate polydispersity index (PDI) of 0.2 (< 0.3). An acceptable explanation for this change is that DSL evaluates the hydrodynamic radius of the colloidal particles, so also considers the coordinate molecules of solvent. The NPs size measured using TEM is referred only to the inorganic core 29. Numerous studies have confirmed that metallic NPs exhibit different tumor-targeting abilities depending on their size. NPs sized less than 20 nm in size exhibited low tumor-specific accumulation. NPs with sizes greater than 300 nm are easily eliminated by phagocytosis. Therefore, NPs with a size of approximately 200 nm are advantageous for tumor accumulation 30. Taken together, these results suggest that CJ-AuNPs may be advantageous for cancer therapy.

**Figure 3:**
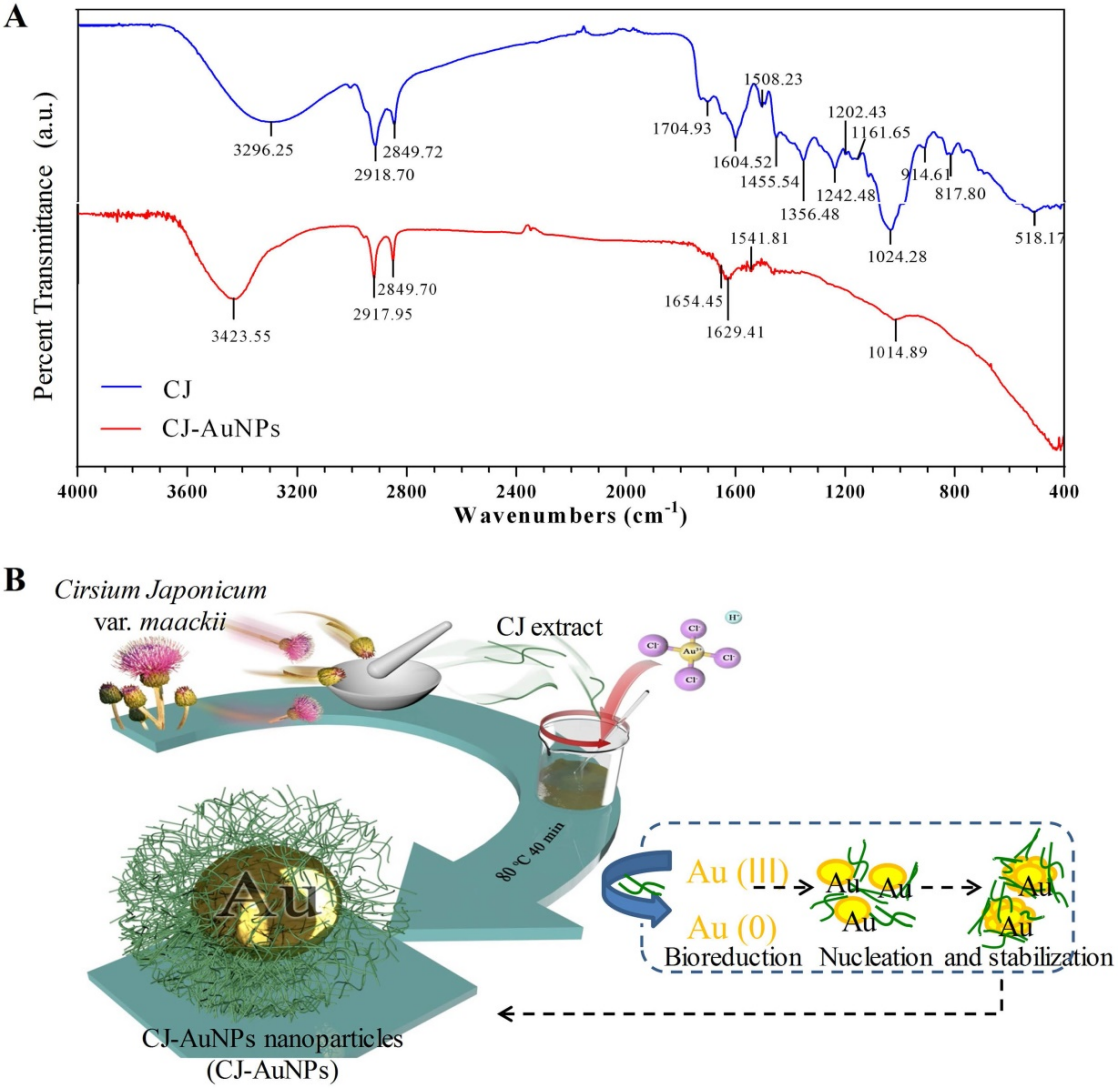
The fourier-transform infrared (FT-IR) spectra of CJ extract and CJ-AuNPs **(A)**. The proposed mechanism for the formation of AuNPs synthesized using CJ extract **(B)**.

The fourier-transform infrared (FT-IR) spectroscopy was employed to determine the possible chemical functional groups responsible for the reduction and stabilization of gold ions during NPs synthesis. The FT-IR spectra of the CJ ethanol extract and CJ-AuNPs were shown in Fig. 3A. The CJ-AuNPs exhibited slightly different absorption patterns and shifted from the CJ. The stretching observed at 3296.2 cm^-1^ (CJ) and 3423.5 cm^-1^ (CJ-AuNPs) corresponded to the O-H groups functional group of phenolic compounds or alcohols 31. A slight shift occurred, indicating that the O-H groups of CJ were capped and stabilized. Likewise, the peak bands at 2918.7, 2849.7 cm^-1^ for CJ and 2917.9, 2849.7 cm^-1^ for CJ-AuNPs were corresponded to the C-H stretching. The characteristic peaks of CJ and CJ-AuNPs at 1704.9-1508.2 cm^-1^ represented the C=O and C=C vibrations in the carbonyl group, confirming the presence of flavonoids in CJ and CJ-AuNPs 19. Previous studies have reported the roles of the O-H and C=O groups of terpenoids, flavonoids, flavones, and phenolic compounds as capping, reducing, and stabilizing agents for NPs green synthesis 5. From above results, we verified the reduction of gold ions by CJ phytochemicals and the formation of an organic coating on the AuNPs surface, which could enhance their biological effects. We propose a mechanism for the formation of AuNPs as shown in Fig. 3B. The CJ phytochemicals reduce the Au^3+^ into Au^0^, which leads to the formation of nuclei. The nuclei coalesce and contribute to the growth of AuNPs 32. Flavonoids with O-H and C=O groups, such as cirsimarin can bind to AuNPs surfaces and act as a stabilizing agent.

### Stability of CJ-AuNPs

**Figure 4:**
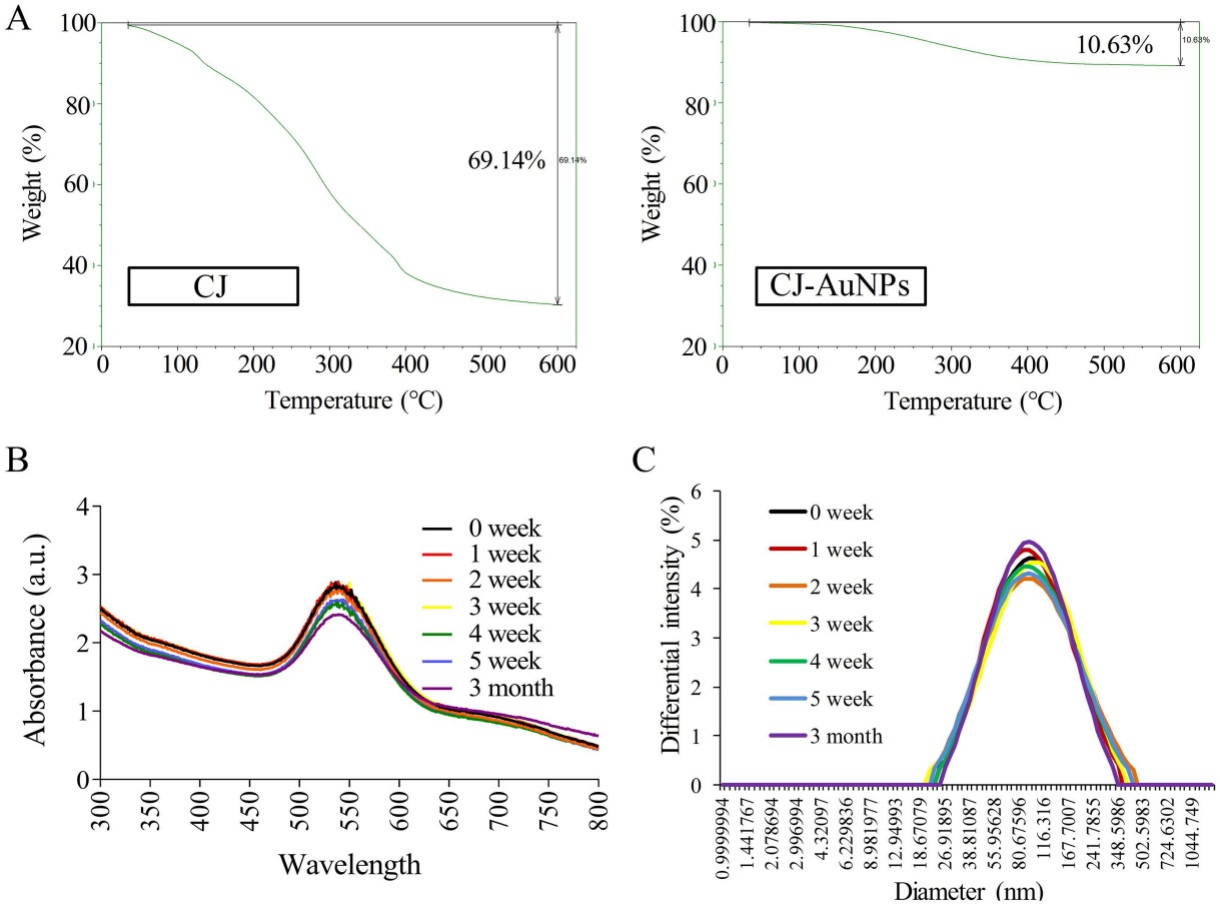
CJ-AuNPs stability. The thermal stability of CJ and CJ-AuNPs at a temperature range of 0-600°C using TGA analysis **(A)**. UV-Vis absorption spectroscopy analysis **(B)** and DLS measurements **(C)** of CJ-AuNPs after 0, 1, 2, 3, 4, 5 weeks, and 3 month from the day of preparation.

The stability of NPs is essential parameter in biological applications. This parameter should be considered when synthesizing nanomaterials with potential industrial applications 33. Fig. 4A shows the thermogravimetric analysis (TGA) results, which indicate the stability of CJ and CJ-AuNPs in the temperature range of 0-600 °C. The degradation analysis of the CJ samples revealed that the temperature could be divided into four stages. During the first stage, the weight loss was related to the evaporation of H_2_O present in the sample in the temperature range 0-100 °C. In the second stage, the weight loss is related to the thermal depolymerization of aryl ether bonds in various constituents of the sample, which occurs at a temperature of 100-400 °C. This stage reached the maximum decomposition temperature, which has been defined in the literature as the corresponding temperature of the maximum decomposition rate 34. The C-C linkages were decomposed when the temperature was above 400-600 °C. The decomposition rate of the sample has started to slow down at this point. When the temperature is above 600 °C, the decomposition of organic groups in CJ was basically complete, and the system has constant weight, CJ extract degraded with a great degree (69.1%). The degradation analysis of the CJ-AuNPs samples was also shown in Fig. 4A (the right). Since Au metal remains morphologically stable below 1000 °C, the thermal weight loss (10.6%) shown in this figure is the thermal weight loss of CJ attached to the surface of Au metal. Therefore, it is calculated that the formation of organic polymer coating on the surface of CJ-AuNPs, the content of CJ molecules in CJ-AuNPs is 15.37%. These results suggest that the synthesized CJ-AuNPs have better thermal stability than CJ.

UV-Vis spectroscopy is the most commonly used technique for characterizing NPs and confirming their formation and stability 9. Therefore, the long-term stability of CJ-AuNPs was assessed by recording the electronic absorption spectra after 1, 2, 3, 4, 5 weeks, and 3 months from the day of preparation using same CJ-AuNPs. As shown in Fig. 4B, CJ-AuNPs demonstrated high stability in PBS within 3 month. Furthermore, the stability of CJ-AuNPs was investigated by measuring particle size as a function of time. The particle sizes of CJ-AuNPs remained stable within 3 months, indicating their stability in aqueous media (Fig. 4C). Moore *et al*. reported that the coating process of AuNPs occurs through multipoint attachment, which helps stabilize the NPs and prevents their agglomeration, which may account for the high stability of CJ-AuNPs 35. These results suggest that the generated CJ-AuNPs were highly stable, which promotes their pharmacological action through contact with and internalization into target cells.

### Cytotoxic Effect of CJ-AuNPs against Normal and Cancer Cells

The biocompatibility of AuNPs is a critical factor affecting their biomedical application, and cytotoxicity to normal cells limits their therapeutic potential 36. Therefore, we evaluated the cytoxicity of CJ-AuNPs in RBC and normal mammalian cell lines, such as RAW264.7 macrophages, normal human dermal fibroblasts (NHDF) cells, and human embryonic kidney 293 (HEK293) cells. As shown in Fig. S1, the percentage of hemolysis of RBC were found to be 1.4%, 3.4%, 4.5%, 5.1%, and 6.3% upon exposure to CJ-AuNPs with concentrations of 20, 40, 80, 100, and 150 µg/mL, respectively. By comparison, CJ extract showed only minor hemolysis (less than 3%) to RBC. No more than 10% of hemolysis was observed for all tested concentrations, which is below the safe hemolytic ratio for biomaterials according to ISO/TR7406 37. These results suggested that CJ-AuNPs was hemocompatible material allowing future biomedical applications. Furthermore, when evaluated at different concentrations (6.25-200 μg/mL), CJ-AuNPs had no significant inhibitory effect on normal cell lines (Fig. S2A). In contrast, CJ-AuNPs (100, 150, and 200 μg/mL) treatment strongly reduced AGS cell viabilities by 23.7-70.7% in a dose-dependent manner, compared to untreated cells (Fig. S2B). These results suggest that CJ-AuNPs could have an anti-cancer activity in AGS cells *in vitro* without induce significant toxic effects on normal cells. The active compounds in CJ extract, as capping agents, might be the main reason for the biocompatibility of CJ-AuNPs in normal cells and anticancer activity in cancer cells.

**Figure 5:**
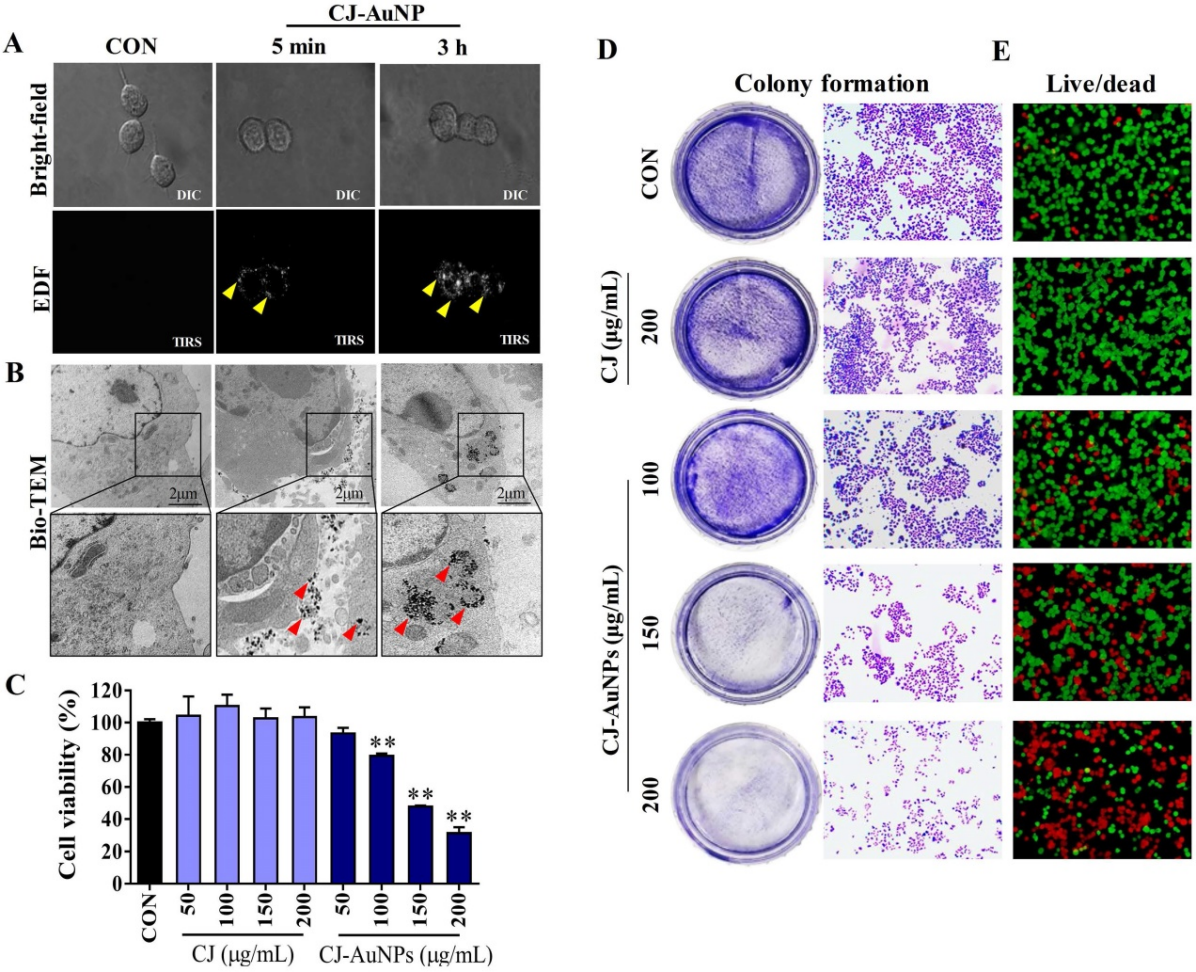
*In vitro* anticancer studies of biosynthesized CJ-AuNPs. Uptake of CJ-AuNPs in AGS cells by enhanced dark-field (EDF) microscopic images of CJ-AuNPs-treated AGS cells **(A)**; Subcellular localization of CJ-AuNPs assessed in AGS cells using Bio-TEM imaging **(B)**; Comparison of cytotoxicity of CJ and CJ-AuNPs in AGS cells **(C)**; Colony formation of AGS cells **(D)**; Fluorescence images of AGS cells stained with live/dead dyes. Red coloring indicates dead cells, and green coloring indicates live cells **(E)**. All values were expressed as mean ± S.D. ** *p* < 0.01 *vs*. untreated group.

Enhanced dark-field (EDF) microscopy was used to observe the cellular uptake of the CJ-AuNPs in AGS cells. As shown in Fig. 5A, CJ-AuNPs (150 μg/mL) rarely accumulated inside the cells after 5 min of treatment. However, after 3 h of incubation, aggregated bright white spots (indicated by the yellow arrows) were observed inside the cells, indicating a rapid increase in the uptake of CJ-AuNPs (Fig. 5A). In contrast, the untreated cells did not exhibit any trace of uptake. The subcellular localization of CJ-AuNPs in AGS cells was investigated using Bio-TEM (Fig. 5B). Consistently, CJ-AuNPs were taken up into the cells after incubation for 3 h and were mainly detected in endosomes in relatively large and dense aggregates, as indicated by black spots inside the cells. These results reflect that the synthesized CJ-AuNPs can be taken up by AGS cells, which may achieve further behaviors of intracellular trafficking of CJ-AuNPs. We further evaluated the anticancer effects of CJ-AuNPs in AGS cells. A commercial MTT assay was performed to assess and compare the cytotoxic effects of CJ extract and CJ-AuNPs. As shown in Fig. 5C, CJ did not exhibit any cytotoxicity in AGS cells, whereas CJ-AuNPs significantly decreased cell viability in a dose-dependent manner. Furthermore, we observed a strong inhibition on the colony formation in AGS cells in response to CJ-AuNPs treatment (Fig. 5D). Fig. 5E shows the representative microscopic images of live/dead staining. The results revealed that CJ did not induce widespread cell death, but large number of cells died following CJ-AuNPs treatment, which reflects the MTT result. Taken together, these results suggest that CJ-functionalized AuNPs can be rapidly taken up by cells and subsequently exhibit a considerable antitumor effect in AGS cells *in vitro*.

### CJ-AuNPs triggers oxidative, iron-dependent cell death

**Figure 6:**
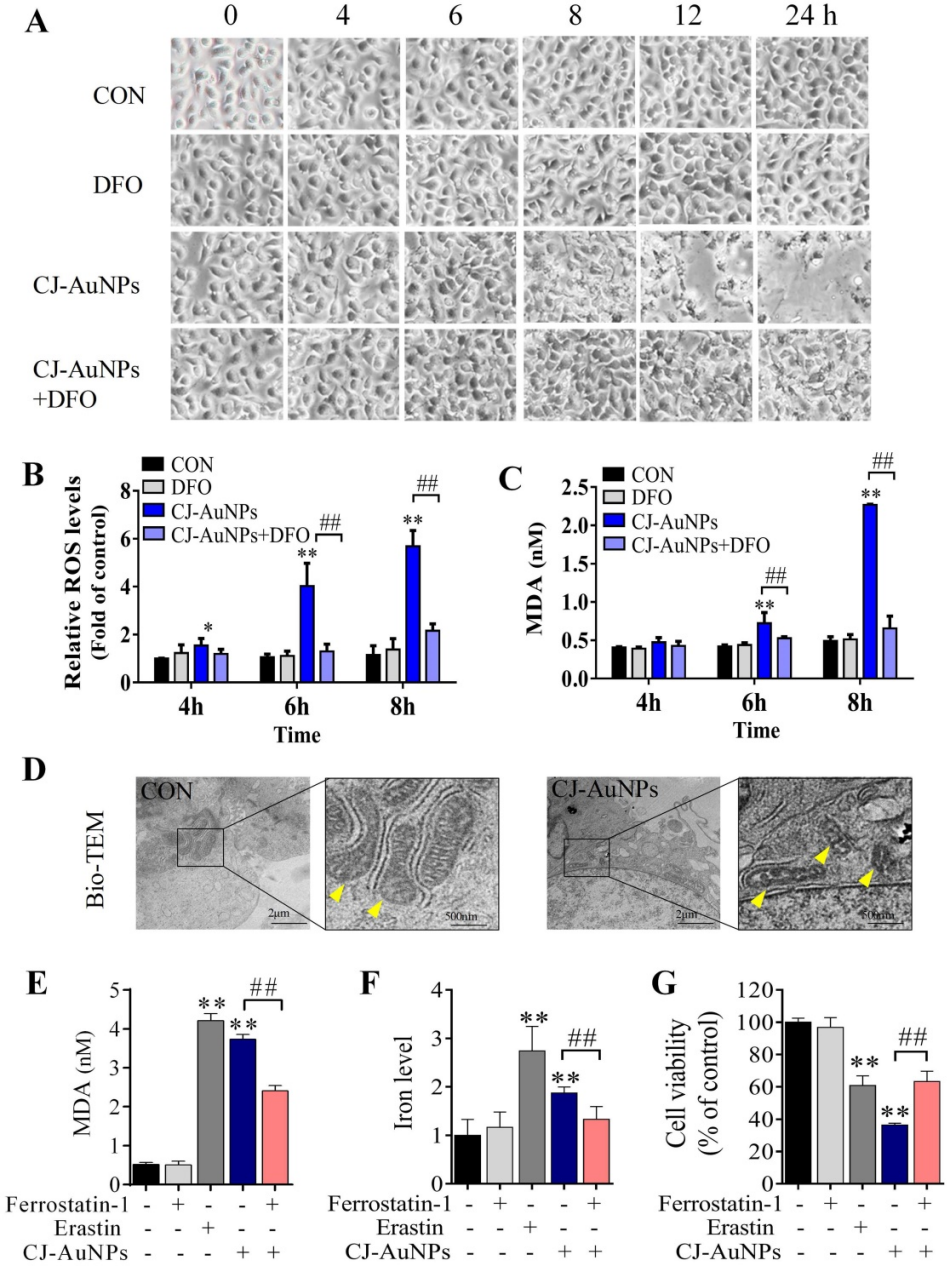
** CJ-AuNPs triggers oxidative and iron-dependent cell death.** Visualization of AGS cells viability over time with and without CJ-AuNPs (150 µg/mL) and deferoxamine (DFO, 100 µM) **(A)**; Cellular ROS production **(B)** and the intracellular malondialdehyde (MDA) concentration **(C)** assessed over time (4, 6, and 8 h); Bio-TEM imaging of AGS cells from untreated and CJ-AuNPs-treated groups. Yellow arrowheads indicate mitochondria **(D)**; MDA content **(E)** and iron level **(F)** measurement in AGS cells; Effect of ferrostatin‐1 (2 µM) on CJ-AuNPs treated AGS cells viability **(G)**. Data are expressed as the mean ± S.D. * *p* < 0.05, ** *p* < 0.01 *vs*. untreated group; ## *p* < 0.01 *vs*. CJ-AuNPs-treated group.

In general, reactive oxygen species (ROS) play a critical role in various types of cell death, including apoptosis, autophagy, and ferroptosis. This fundamental conserved mechanism is based on the attack biomembranes by excess ROS, which triggers lipid peroxidation chain reactions to induce various types of cell death 38. Interestingly, ROS are considered a major inducement of cell death caused by toxic NPs 20. Hence, we investigated whether CJ-AuNP-induced cytotoxicity was associated with oxidative stress damage in AGS cells. As shown in Fig. 6B, CJ-AuNPs (150 µg/mL)-treated AGS cells showed a time-dependent increase in cytosolic ROS beginning at 4 h. This increase in ROS preceded cell detachment and overt cell death, which began at 8 h (Fig. 6A). Cell death occurred in the CJ-AuNPs-treated cells following a prolonged period of ROS accumulation. Some reports have shown that ROS-induced lipid peroxidation plays an important role in different types of cell death including apoptosis and ferroptosis. This conserved mechanism is based on an excess of ROS which attacks the biomembrane, triggers lipid peroxidation chain reaction, and subsequently induces cell death 38,39. MDA, the final lipid peroxidation product, is often used as a lipid peroxidation marker 40. Our results showed that CJ-AuNPs increased MDA levels in AGS cells in a time-dependent manner (Fig. 6C). Interestingly, CJ-AuNPs-induced ROS accumulation, lipid peroxidation, and cell death were reversed after co-treatment with deferoxamine (DFO, 100 µM), a well-known iron chelator specifically used in iron overdoses, by chelating ferrous ions and inhibiting ferroptosis (Fig. 6A-C) 41. These results revealed that the overwhelming iron-dependent ROS accumulation and lipid peroxidation are responsible for inducing cell death by CJ-AuNPs. In recent years, a new form of programmed cell death, known as ferroptosis, has been described. It is caused by ROS and lipid peroxidation, which highlights the role of lipid peroxidation in programmed cell death 41. A recent study reported that salinomycin-loaded AuNPs induced iron accumulation and inhibited antioxidant properties in cancer stem cells, thereby leading to iron-dependent cell death 42. Similarly, the results obtained in the present study for the CJ-AuNPs may suggest a ferroptosis. Therefore, it is of great interest to explore whether ferroptosis is a mechanism of cell death induced by CJ-AuNPs.

### CJ-AuNPs induce mitochondrial dysfunction and intracellular ferrous iron overload in AGS cells

Cancer cells often have defects in cell death executioner mechanisms, which is one of the main reasons leading to drug resistance. To enable growth, cancer cells exhibit a higher iron demand and dependency than normal cells, which make cancer cells more vulnerable to iron-catalyzed necrosis, referred to as ferroptosis 43. A previous study reported that metal NPs could impair mitochondria by bursting ROS production, disrupting iron metabolism, eliciting lipid peroxidation, and ultimately triggering ferroptosis 20. Mitochondrial ultrastructural changes are the typical characterizatics of ferroptosis, such as condensed mitochondria, increased membrane density, and cristae disappearance 41. In this study, we observed normal mitochondrial morphology in AGS cells in the control group, characterized by normal mitochondrial cristae and a complete membrane structure. However, AGS cells treated with CJ-AuNPs showed smaller mitochondria, accompanied by increased membrane density and fused mitochondrial cristae (Fig. 6D). Kloditz *et al*. also observed cell morphology in ferroptosis using Bio-TEM, and successfully differentiated ferroptotic cells by mitochondrial changes similar to those observed in this study 44. This result indicated that CJ-AuNPs may trigger ferroptosis in AGS cells.

To further confirm the role of CJ-AuNPs in ferroptosis induction in AGS cells, ferrostatin-1, a well-known ferroptosis inhibitor, was used to prevent CJ-AuNPs-induced ferroptosis. The feature of erastin (a ferroptosis inducer)-triggered ferroptosis is excess lipid peroxidation induced by the accumulation of intracellular lipid ROS in an iron-dependent manner 41. Nanomaterial-mediated ferroptosis exhibits the same hallmarks as erastin, such as lipid peroxidation and iron overloading 20. Therefore, we assessed erastin- and CJ-AuNPs-induced changes in lipid peroxidation and intracellular Fe^2+^ in AGS cells. Malondialdehyde (MDA), a lipid peroxide product, was also investigated. Consistent with the effect of erastin, CJ-AuNPs significantly induced MDA production and intracellular iron overload, which correlated with ferroptosis sensitivity (Fig. 6E-F). However, the increased MDA content and Fe^2+^ overload caused by CJ-AuNPs were significantly reduced in the presence of ferrostatin-1 (2.0 μM) in AGS cells. Furthermore, ferrostatin-1 partially restored cell viability decreased by CJ-AuNPs, suggesting that CJ-AuNPs-induced cell death can be reversed by inhibiting ferroptosis in AGS cells (Fig. 6G). These results demonstrated that the anticancer effect of CJ-AuNPs in AGS cells was achieved, at least in part, by inducing ferroptosis. Consistent with our results, Zhao *et al*. found that salinomycin-loaded AuNPs induced ferroptosis-related cancer cell death by increasing lipid peroxidation and Fe^2+^ accumulation 42. However, we note that in this study the Tween 20 was used for salinomycin-AuNPs synthesis. Tween 20 is biologically lethal (ADI 0-25 mg/kg (FAO/WHO, 2004), LD50 37 g/kg (rat, oral)), which undoubtedly makes the biological application of this product in humans difficult 45. Therefore, we prepared CJ-AuNPs with the similar anticancer mechanism using the green material CJ as a bioreductive agent, which eliminated the influence of toxic chemical material on biological application and provided advancement for practical application of the final product.

### CJ-AuNPs induces ferroptosis via regulating GPX-4 and HO-1

**Figure 7:**
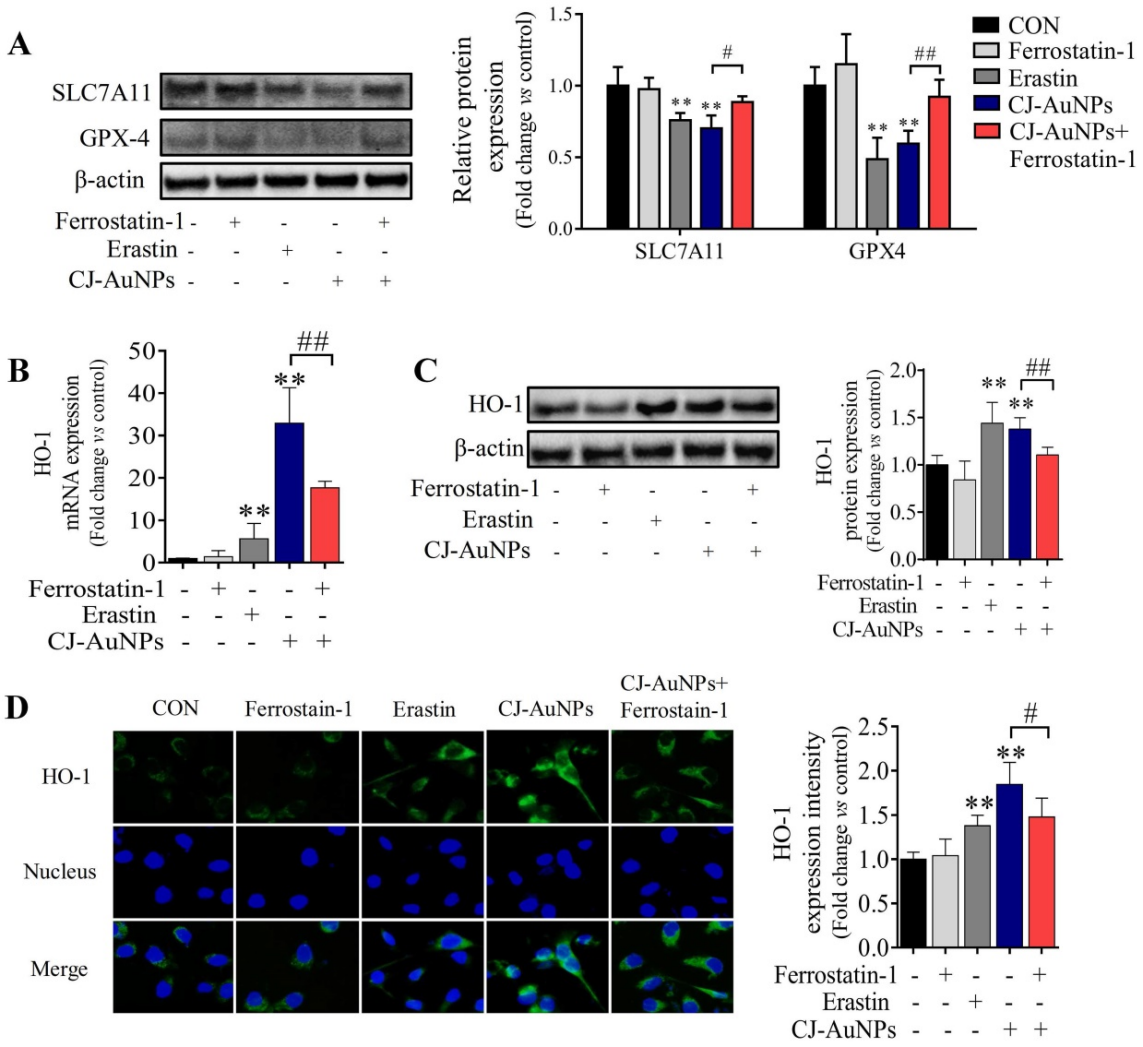
** CJ-AuNPs induces ferroptosis by regulating GPX-4 and HO-1.** Effect of ferrostatin‐1 (2 µM) on CJ-AuNPs (150 µg/mL) upregulated SLC7A11 and GPX-4 protein expressions in AGS cells **(A)**; Ferrostatin‐1 (2 µM) partially reversed the upregulation of HO-1 mRNA expression **(B)** and protein expression **(C)** in CJ-AuNPs (150 µg/mL) treated AGS cells. The protein expression of HO-1 was detected by immunofluorescence analysis in AGS cells. The stained cells (green color) were recorded with a fluorescence microscope **(D)**. All protein expression analyzed by immunoblot were standardized by β-actin. Data are expressed as the mean ± S.D. * *p* < 0.05, ** *p* < 0.01 *vs*. untreated group; # *p* < 0.05, ## *p* < 0.01 *vs*. CJ-AuNPs-treated group.

To determine the molecular mechanism of CJ-AuNPs-triggered ferroptosis, we detected the expression of antioxidant/ferroptosis-related proteins using western blotting. Although the mechanisms underlying the regulation of ferroptosis remain poorly understood, the core mechanism of lipid peroxidation has been well documented. Lipid peroxidation can drive ferroptosis, which is typically triggered by suppressing solute carrier family 7 member 11 (SLC7A11) and glutathione peroxidase 4 (GPX-4) 46. SLC7A11 is a multipass transmembrane protein that mediates cystine/glutamate antiporter activity in system x_c_^-^. Inhibiting SLC7A11 expression can deplete cystine, consequently curtailing glutathione and increasing lipid peroxidation 47. GPX-4, an antioxidant defense enzyme, can reduce complex hydroperoxides to their corresponding counterparts, neutralizing the chain reaction of lipid peroxidation and thereby acting as a critical inhibitor of lipid peroxidation. GPX-4 downregulation can facilitate the accumulation of lipid peroxides, which are the primary determinants of ferroptosis 46. To verify the effect of ferroptosis, protein markers were detected in nearly all studies, especially SLC7A11 and GPX-4 suppressions 20. Zhao *et al*. confirmed that salinomycin-AuNPs could effectively induce ferroptosis by inhibiting GPX-4 protein expression, whereas salinomycin showed no effect, suggesting a possible target for AuNPs-induced ferroptosis 42. In this study, we measured the expression levels of SLC7A11 and GPX-4 proteins in CJ-AuNPs-treated AGS cells and found that erastin markedly reduced SLC7A11 and GPX-4 expression (Fig. 7A). Under the same experimental conditions, SLC7A11 and GPX4 expression levels were significantly inhibited in CJ-AuNPs-treated AGS cells, suggesting that the core mechanism of ferroptosis was triggered by CJ-AuNPs. Consistent with our results, Rossi *et al*. reported that partial embedding of AuNPs across the membrane could cause ferroptosis-related protein dysfunction in the membrane and ultimately trigger ferroptosis 48.

To further understand the mechanism of action of the CJ-AuNPs, the expression of heme oxygenase 1 (HO-1), an essential enzyme in heme catabolism, was evaluated using qRT-PCR (Fig. 7B), immunoblotting (Fig. 7C), and immunofluorescent staining analyses (Fig. 7D). Interestingly, CJ-AuNPs considerably increased the expression of gene- and protein-encoding HO-1, while ferrostatin-1 significantly reversed CJ-AuNPs-induced HO-1 upregulation (Fig. 7C-D). HO-1 protects cells against various stress-related conditions by metabolizing heme into biliverdin/bilirubin, carbon monoxide, and ferrous iron 49. Recently, the non-canonical ferroptosis induction function of HO-1 has shown that HO-1 activation can trigger heme degradation and in turn release Fe^2+^, which is sufficient to induce ferroptosis through free iron accumulation-induced lipid peroxidation 49,50. Consistent with our results, Hassannia *et al*. demonstrated that withaferin A induce ferroptosic cell death in neuroblastoma by increasing intracellular Fe^2+^ levels upon the excessive activation of HO-1 51. These results indicated that the CJ-AuNPs-mediated ferroptosis by targeting GPX-4 and enhancing the Fe^2+^ pool through excessive HO-1 activation might open new perspectives for the development of novel treatments for cancer cell death.

### *In vivo* antitumor activity of biosynthesized CJ-AuNPs

As an innovative clinical research approach, nanotechnology has been reported by Hainfeld *et al*. to induce smaller tumor size and higher survival rates in mice treated with AuNPs 52. Likewise, other studies have revealed that AuNPs might have significant therapeutic potential for patients with post-cancer metastases 53. Accordingly, considering the significant anti-GC activity of CJ-AuNPs in AGS cells *in vitro*, we investigated whether CJ-AuNPs-induced treatment responses could be generated in AGS gastric adenocarcinoma xenograft models. Therefore, we established a xenograft mouse model with AGS cell implantation. 5-FU was also administered to mice as a positive control to determine the safety and anticancer efficacy of CJ-AuNPs.

**Figure 8:**
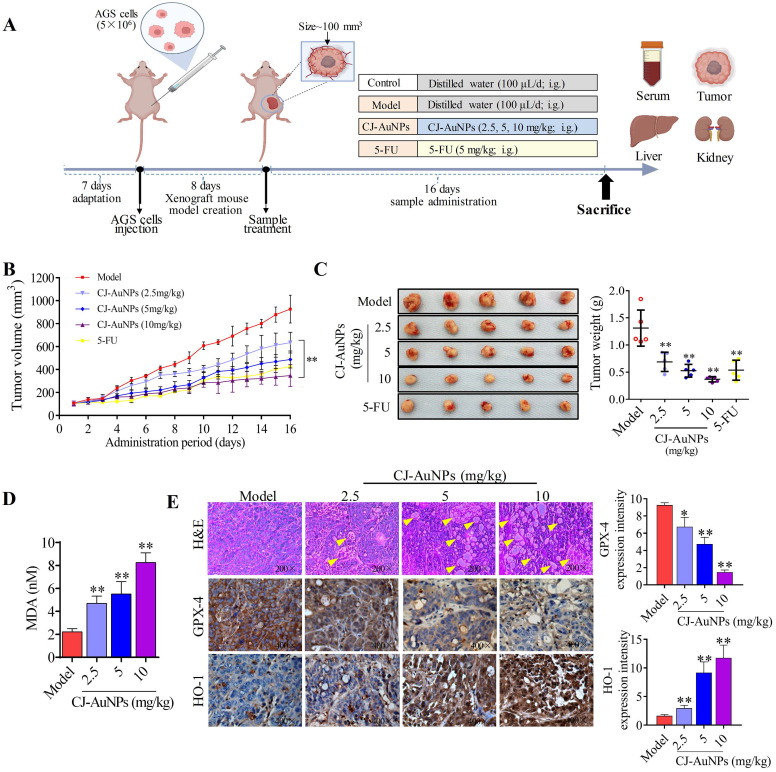
***In vivo* antitumor activity of biosynthesized CJ-AuNPs.** Experimental design *in vivo* of this study **(A)**; Change in tumor volume during treatment period **(B)**; Tumor photos and tumor weights after treatment **(C)**; The effect of CJ-AuNPs on MDA content in tumor tissue **(D)**; hematoxylin and eosin (H&E) staining of tumor slices obtained from AGS xenograft-bearing mice (**E,** up), the yellow arrows indicated the formation of cavities; immunohistochemical staining of GPX-4 (**E,** middle) and HO-1 (**E,** down) in tumor tissues, and positive signal analysis of immunohistochemistry (right). Data are expressed as the mean ± S.D. * *p* < 0.05, ** *p* < 0.01 *vs*. model group.

After establishing the xenograft mouse model implanted with AGS cells, each group of mice was administered different medications, as shown in Fig. 8A. The tumor volume of mice in each group was recorded throughout the administration period to determine the effects of CJ-AuNPs on tumor progression. As expected, relative to the rapidly increasing tumor volumes measured after oral administration of the saline vehicle (tumor control), significant tumor inhibition was observed with 5-FU (Fig. 8B). Daily intake of CJ-AuNPs at doses of 2.5, 5, and 10 mg/kg also markedly and dose-dependently decreased tumor volume by 31.59%, 47.45%, and 62.49%, respectively, compared to that in model mice (Fig. 8B). The visual inspection of the tumor tissues was consistent with the change of tumor volume of each group (Fig. 8C). Furthermore, the tumor weight of the mice also decreased after CJ-AuNPs treatment in a dose dependent manner (Fig. 8C). In particular, compared to those in model mice, oral administration of CJ-AuNPs at doses of 10 mg/kg significantly decreased tumor volume and weight by 62.5% and 71.9%, respectively. Similarly, Lee *et al*. 54 suggested that the AuNPs treatment dramatically reduced tumor growth, which agrees with the results of the current study.

To examine whether the inhibition of tumor growth resulting from CJ-AuNPs treatment could be related to ferroptosis, we investigated CJ-AuNPs-induced changes in lipid peroxidation. Consistent with the effect of CJ-AuNPs in AGS cells *in vitro*, CJ-AuNPs induced MDA production in a dose dependent manner, compared with that in the tumors of model mice (Fig. 8D). Furthermore, we investigated the expression of genes and proteins related to the ferroptosis pathways in tumor tissues to verify their roles in tumor development (Fig. 8E). First, H&E staining showed significant tumor cell shrinkage, nuclear rupture, tumor tissue necrosis, and cavity formation (indicated by the yellow arrows) in the CJ-AuNPs-treated groups, indicating that the CJ-AuNPs can effectively induce tumor cell death and tissue damage. Correspondingly, IHC staining revealed that the density of GPX-4 significantly decreased in tumor tissues isolated from CJ-AuNPs-treated mice compared to that in model mice, indicating the occurrence of CJ-AuNPs-induced ferroptosis. HO-1 density significantly increased in a dose-dependent manner (Fig. 8E), the gene expression of HO-1 was also upregulated accordingly (Fig. S3), which is consistent with the results obtained in our cell experiment. These results validated our *in vitro* finding that CJ-AuNPs exert anticancer effects *via* ferroptosis-mediated cancer cell death, suggesting that ferroptosis is at least partly responsible for CJ-AuNPs-induced tumor suppression *in vivo*.

### *In vivo* toxic effects of the biosynthesized CJ-AuNPs

Biosynthesized AuNPs have been considered as anticancer candidates 55. However, most reports showed significant anticancer activity of biosynthesized AuNPs *in vitro*, and studies should assess potential toxic effects of biogenic AuNPs through *in vivo* studies, which was inadequate from such studies 56.

**Figure 9:**
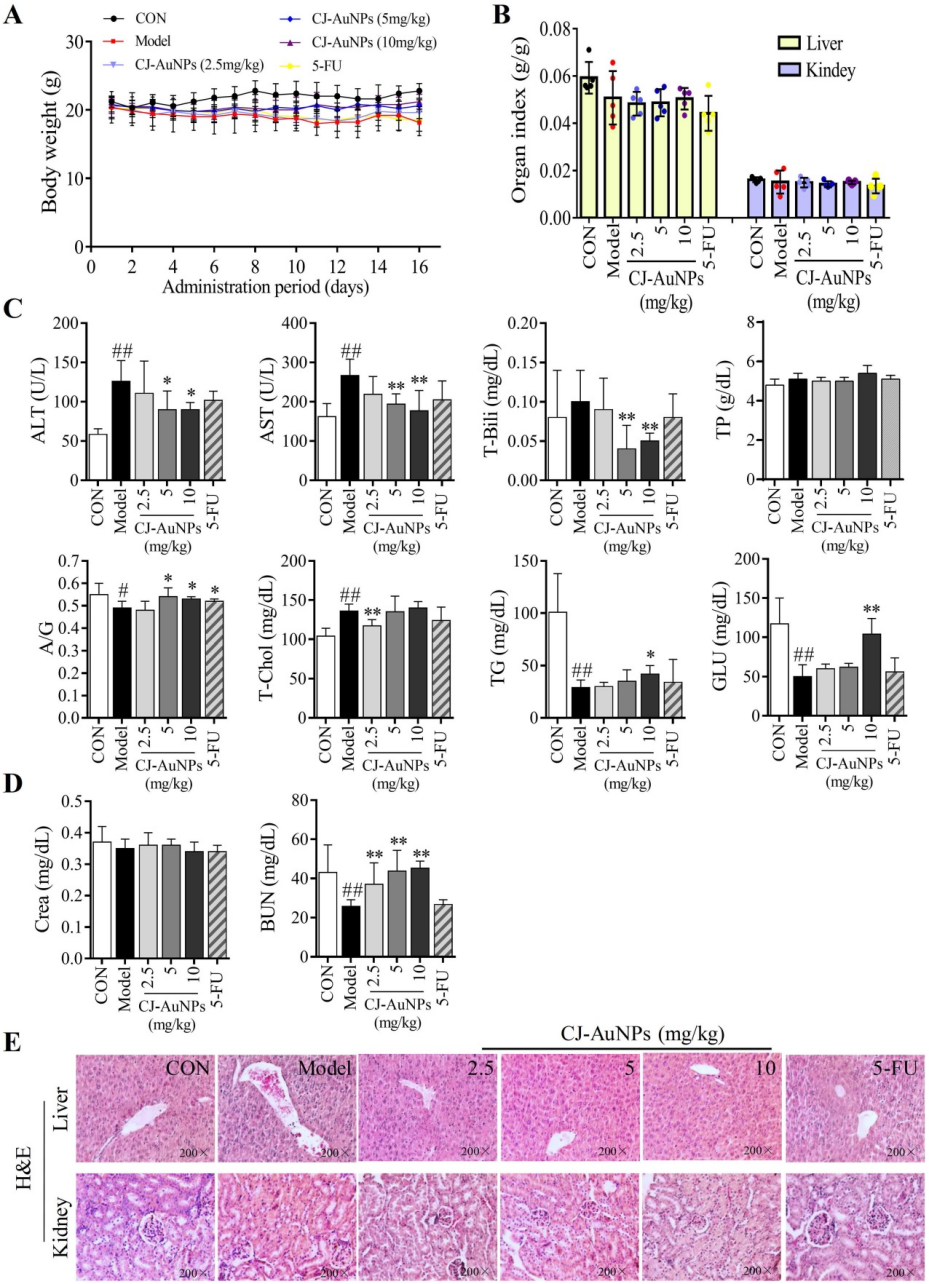
***In vivo* toxic effects of the biosynthesized CJ-AuNPs.** Change in body weight during treatment period **(A)**; The organs index of liver and kidney in control mice and xenograft-bearing mice **(B)**; Detection of serum ALT (U/L), AST (U/L), T-Bili (mg/dL), TP (g/dL), A/G ratio, T-Chol (mg/dL), TG (mg/dL), and GLU (mg/dL) levels **(C)**; Detection of serum Crea (mg/dL) and BUN (mg/dL) levels **(D)**; The histopathological observations of liver and kidney in mice using hematoxylin and eosin (H&E) staining **(E)**. Data are expressed as the mean ± S.D. #p<0.05, ##p<0.01 *vs*. control group. * *p* < 0.05, ** *p* < 0.01 *vs*. model group. ALT: alanine aminotransferase; AST: aspartate aminotransferase; T-Bili: total bilirubin; TP: total protein; A/G: serum albumin/globulin; T-Chol: total cholesterol; TG: triglyceride; GLU: glucose; Crea: creatinine; BUN: blood urea nitrogen.

To evaluate the toxicity effects of CJ-AuNPs, we recorded body weight throughout the administration period. As shown in Fig. 9A, the mice in the control group showed a steady increase in body weight. No statistically significant differences were observed in the body weight between the model and CJ-AuNPs-treated groups. However, body weight was lower in the 5-FU-administered group than that in the CJ-AuNPs-treated group, which is consistent with previous reports on the side effects of 5-FU^57^. Moreover, no significant differences were observed in the liver and kidney indices between the groups (Fig. 9B). The toxic effects of CJ-AuNPs were further assessed *in vivo* by measuring various biochemical parameters. Liver function indices including alanine transaminase (ALT), aspartate aminotransferase (AST), total bilirubin (T-Bili), total protein (TP), albumin/globulin (A/G) ratio, total cholesterol (T-Chol), triglyceride (TG), and serum glucose (GLU) were measured. ALT and AST levels are indicators of proper liver function. Damage to the liver tissue may lead to a disturbance in the amount of these enzymes secreted into the bloodstream. As shown in Fig. 9C, ALT, AST, and T-Chol levels in the model group were considerably higher than those in the control group. The serum A/G ratio, TG, and GLU levels were significantly lower than those in the control group. This illustrates that the AGS-xenograft tumor affected liver function in mice. However, CJ-AuNPs (2.5, 5, and 10 mg/kg)-treated mice displayed lower levels of ALT and AST than the model group in a dose-dependent manner, indicating relatively healthy hepatic cells, which is in agreement with the results reported by Jo *et al.* 58. Compared to the model group, CJ-AuNPs also decreased the levels of T-Chol and T-Bili by 14% and 50% at low (2.5 mg/kg) and high (10 mg/kg) concentrations, respectively (Fig. 9C). Although there was no statistically significant difference between the control, model, and CJ-AuNPs (2.5, 5, and 10 mg/kg)-treated groups in terms of TP level, the CJ-AuNPs (10 mg/kg) significantly reversed the decrease in the A/G ratio, compared to the model group, which suggests that CJ-AuNPs may have a protective effect in the liver (Fig. 9C). Although these biochemical indicators in the 5-FU group were higher or lower than those in the model group, observed differences were not statistically significant. These results suggest that CJ-AuNPs were not toxic to the liver and showed hepatoprotective effects against AGS xenograft-induced liver dysfunction. Furthermore, renal function indices including creatinine (CRE) and blood urea nitrogen (BUN) of all mice were detected, and the results are presented in Fig. 9D. There was no significant difference in the level of CRE in the control, model, and CJ-AuNPs groups. The BUN levels of model group (25.8±3.3 mg/dL) are significantly lower than those of the control group (43.1±14.1 mg/dL), which was restored to 37.1±10.9, 43.7±10.7, and 45.3±3.6 mg/dL by CJ-AuNPs treatment at the concentrations of 2.5, 5, and 10 mg/kg, respectively. This illustrated that CJ-AuNPs were not toxic to the kidney and showed a protective effect against AGS xenograft-induced kidney dysfunction.

The toxic effects of the CJ-AuNPs were further evaluated by the histopathological observations of liver and kidneys. As shown in Fig. 9E, the liver of control group had normal histology with hepatocytes and a central vein, while the model group exhibited swollen morphology and poorly formed central vein with mononuclear cell infiltration in the hepatocytes. Nevertheless, these pathologic changes were relieved after treatment with CJ-AuNPs, and levels in the high dose (10 mg/kg) group almost returned to normal (Fig. 9E). The kidneys revealed normal histology of white and red pulp, and no histological differences in the kidneys were observed in the CJ-AuNPs treatment groups, indicating no notable toxicity (Fig. 9E). Overall, these results indicated that CJ-AuNPs and 5-FU were not toxic to the kidneys or liver. More importantly, compared to 5-FU, CJ-AuNPs showed a protective effect against AGS xenograft-induced kidney and liver dysfunction, suggesting the safety of CJ-AuNPs in cancer treatment as a novel anti-GC drug candidate.

## Conclusion

This study aimed to develop a green technique for synthesizing AuNPs from the *Cirsium japonicum* extract and further encourage biomedical applications of AuNPs as anticancer drugs. The optimal conditions for CJ-AuNPs biosynthesis were monitored, and the physicochemical structures of CJ-AuNPs were determined using various microscopic and spectrometric analyses. The biological effects of CJ-AuNPs were observed in human gastric cancer AGS cells *in vitro*, confirming the activation of ferroptosis. First, system xc- and GPX-4 inhibition contributed to lipid peroxidation during ferroptosis. Second, the CJ-AuNPs-upregulated HO-1 conferred ferroptosis by increasing iron accumulation. These results suggested that CJ-AuNPs inhibited cell proliferation by triggering ferroptosis-related cell death in AGS cells* in vitro*. In a xenograft mouse model implanted with AGS cells (*in vivo*), CJ-AuNPs markedly restrained tumor growth but did not exhibit significant systemic toxicity. The cytotoxic effect of CJ-AuNPs against GC may be correlated with ferroptosis-mediated cell death and tumor suppression. These results provide preliminary data for the clinical application of CJ-AuNPs as novel anti-GC drug candidates. Ferroptosis inducers, represented by biosynthesized AuNPs, may benefit patients with GC as an alternative to chemotherapy, providing directions for cancer treatment.

## Supplementary Material

Supplementary methods, figures and table.

## Abbreviations

AuNPs: gold nanoparticles
CJ-AuNPs: Cirsium japonicum mediated-AuNPs
ROS: reactive oxy-gen species
GPX4: glutathione peroxidase-4
GC: gastric cancer
DMEM: Dulbecco's modified Eagle's medium
RPMI: Roswell Park Memorial Institute (RPMI)-1640 medium
FBS: fetal bovine serum
HAuCl_4_•3H_2_O: hydrogen tetrachloroaurate hydrate
SEM: means ± standard error
FE-TEM: field emis-sion-transmission electron microscopy
FE-SEM: field emission-scanning electron microscopy
DLS: dynamic light scattering
EDX: energy dispersive X-ray
XRD: the X-ray diffraction
FT-IR: the fourier-transform infrared
TGA: the thermogravimetric analysis
EDF: enhanced dark-field
MDA: malondialdehyde
SLC7A11: solute carrier family 7 member 11
HO-1: heme oxygenase 1
ALT: serum alanine aminotransferase
AST: aspartate aminotransferase
T-Chol: total cholesterol
A/G: the serum albumin/globulin
TG: triglyceride
GLU: serum glucose
T-Bili: total bilirubin
TP: total protein
CRE: cre-atinine
BUN: blood urea nitrogen
H&E: hematoxylin and eosin
