# Supplementary material for: Biologically synthesis of gold nanoparticles using Cirsium japonicum var. maackii extract and the study of anti-cancer properties on AGS gastric cancer cells
Source: Int J Biol Sci. 2022 Sep 21;18(15):5809–26. doi: 10.7150/ijbs.77734 (PMC9576503; doi:10.7150/ijbs.77734)
Supplement: Supplementary file 1 — Supplementary methods, figures and table. [file ijbsv18p5809s1.pdf]

Supplementary information 1

Liquid chromatography-mass spectrometry (LC-MS) analysis of the CJ

The CJ ethanol extract (weighing at 1.0 g), as mentioned above, was mixed with 5% DMSO/MeOH and filtered using a 0.22 μm DISMIC-25 cs disposable syringe filter unit (Advantec, Tokyo, Japan). The filtrate was collected and analyzed on the Ultimate3000 system (Thermo Scientific, USA) to process HPLC data. The waters Cortex C18 column (2.1 mm × 150 mm, 1.6 um) was employed for chromatographic separation at a column temperature of 45 ^{o}C. The mobile phase consisted of solvent A (0.1% formic acid in water) and solvent B (0.1% formic acid in acetonitrile) at a 0.3 mL/min of flow rate. The MS detector was interfaced on HPLC device to calculate the peak of fingerprints. LC/MS analysis was performed using mass spectrometer (Triple TOF 5600+, AB Sciex, USA) with electrospray ionization (ESI) source. ESI-MS research analysis conditions were as follows: negative ion mode, temperature 500 ℃, Nebulizer pressure 50 psi, floating ion spray voltage 4.5 kV.

The stability of CJ-AuNPs

The thermal stability of the CJ-AuNPs was measured by thermogravimetric analysis (TGA) (TGA/DSC 1; Yeonjin S-Tech Co., Seoul, Korea) at 30-600 ℃. Furthermore, the *in vitro* stability of manufactured CJ-AuNPs was tested in the presence of phosphate buffer saline (PBS, pH 7.4) (Biosesang, Gyeonggi, Korea). Briefly, CJ-AuNPs (2.0 mg/mL) was dissolved in PBS and stored for 1, 2, 3, 4, 5 weeks, and 3 month at room temperature. We then recorded the change of

corresponding electronic absorbance spectra and hydrodynamic size of CJ-AuNPs to investigate their stability.

Hemocompatibility assay

Defibrinated Sheep Blood samples were obtained from Kisan Bio Co. Ltd. (Seoul, Korea). The blood samples (5.0 mL) was added to PBS (10.0 mL) and centrifuged at 2000 g for 10 min. The red blood cells (RBCs) were washed five times with PBS solution and then removed supernatant. The suspension of purified RBC was prepared in 50 mL of PBS for further study. Herein, RBCs were incubated with distilled water and PBS as the positive (100%) and negative (0%) control, respectively. CJ extract and CJ-AuNPs suspended in PBS solutions with different concentrations were incubated in RBCs suspension at room temperature for 3 h. Finally, the mixed solutions were centrifuged at 13000 rpm for 10 min, and 100 μL of supernatant was transferred to a 96-well plate. The absorbance values at 570 nm were measured using a UV-Vis spectrophotometer (SpectraMax® ABS plus). The percentage of hemolysis was calculated using the following formula: Hemolysis (%) = $((\text{absorbance}_{\text{sample}} - \text{absorbance}_{\text{negative control}}) / (\text{absorbance}_{\text{positive control}} - \text{absorbance}_{\text{negative control}})) \times 100$.

Cell culture and cell viability evaluation

All the cell lines used in this study were obtained from the Korean Cell Line Bank (KCLB, Seoul, Republic of Korea). Hela cell (Cervical cancer cell line), AGS cells (human stomach adenocarcinoma cell line), HT-29 cells (human colon adenocarcinoma cell line), and HepG2 cell (human liver carcinoma cell line) were cultured in RPMI 1640 medium containing 10% FBS (*v*/*v*) and 1%

penicillin-streptomycin (*v*/*v*). NHDF cells (human fibroblasts), HEK293 cells (human Embryonic Kidney 293 cell), and RAW264.7 cells (macrophage cell line) were cultured in DMEM medium (high glucose) containing 10% FBS (*v*/*v*), 1% penicillin-streptomycin (*v*/*v*). The cells were incubated in a humidified incubator with 5% carbon dioxide (CO_2) atmosphere at 37 ℃.

To detect the effect of CJ-AuNPs on cell viability, each cell suspension was seeded in a 96-well plate (1×10^4 cells/well) and stabilized for 24 h. After the cells were washed twice with phosphate-buffered saline (PBS), the fresh medium containing CJ-AuNPs at various concentrations (6.25, 12.5, 25, 50, 100, 150, 200 μg/mL) was added to the cells for 24 h incubation. The cell viability was measured using a conventional MTT solution according to the manufacturer's instruction. The formed formazan crystal was dissolved in DMSO (100 μL/well) and detected at 570 nm using a microplate reader (SpectraMax® ABS plus).

Evaluation of intracellular uptake and localization of CJ-AuNPs

To observe the uptake capacity of CJ-AuNPs, the gastric adenocarcinoma (AGS) cells were treated with CJ-AuNPs (150 μg/mL) for 5 min and 3 h. Cells were washed with PBS and fixed in 4% paraformaldehyde for preparing slides. To monitor the distribution of the CJ-AuNPs within the cells, the slide was observed under a bright-field microscopy (Olympus Optical Co., Ltd., Tokyo, Japan) and enhanced dark-field (EDF) microscopy (CytoViva Inc., Auburn, AL, USA) to take the photographic results.

Furthermore, the uptake and intracellular localization were detected using a Bio-TEM. Briefly, after CJ-AuNPs (150 μg/mL) treatment for 5 min and 3 h, the cell pellets were collected and fixed in 2.5% glutaraldehyde at 4 ℃. The 1% osmium tetroxide was used to post-fix. After gradually dehydrating with concentration gradient ethanol (50, 70, 90, and 100%), samples were embedded in Epon (Sigma-Aldrich, St. Louis, MO). an ultramicrotome (Leica EM UC7, Wetzlar, Germany) was used to make ultrathin sections (70 nm). The sections were then treated according to the standard protocol for Bio-TEM imaging. Finally, the sections were imaged using JEM-1010 TEM (JEOL, Tokyo, Japan) operated at 80 kV.

Colony formation assay and live/dead staining

For the colony-forming assay, AGS cells (2×10^3 cells/well) were plated in a 6-well plate (SPL Life Sciences, Pocheon, Korea) and stabilized for 24 h. After being washed twice with PBS, the cells were incubated in fresh RPMI 1640 culture medium containing samples (100, 150, and 200 μg/mL) for 36 h. After rinsing with PBS, AGS cells were dyed using 0.5% crystal violet solution (Sigma-Aldrich) for 3 min. The cell colony was observed under a Leica DM IRB inverted microscope. Furthermore, to compare the cytotoxic effect of CJ-AuNPs in AGS cells, a live/dead cell staining kit (Thermo Fisher Scientific) was employed according to the manufacturer's instruction. Briefly, AGS cells (2×10^5 cells/well) were cultured in 6-well plates, stabilized for 24 h, and then incubated with CJ-AuNPs for 24 h. Cells were stained by Calcein (detect live cells) and Ethidium Homodimer-1 reagent (detect dead cells) for 30 min. The living cells (green) and dead cells (red) were subsequently visualized using

fluorescence scanning microscope at Ex/Em 494/517 nm (Leica, Wetzlar, Germany).

Reactive oxygen species (ROS) quantification and lipid peroxidation assessment

The intracellular ROS release was detected by using a Cellular ROS/Superoxide Detection Assay Kit (Abcam, Cambridge, UK). AGS cells (2×10^5 cells/well) were seeded in 6-well plates (SPL Life Sciences) and stabilized for 24 h. After the cells were washed twice with PBS, they were treated respectively with fresh RPMI containing different samples for 4, 6, and 8 h. After rinsing twice with PBS, the cells were stained using a cellular ROS assay kit according to the manufacturer's instructions. Following an incubation period of 30 min, the AGS cells-contained coverslips were inspected on the stage of a fluorescent microscope (Leica, Wetzlar, Germany) and quantified using the GraphPad software (Prism 8; San Diego, CA, USA). To detect the level of lipid peroxidation, the intracellular malondialdehyde (MDA) concentration was assessed at 4, 6, 8, and 24 h after samples treatment using a lipid peroxidation (MDA) colorimetric assay kit (Abcam) according to the manufacturer's protocol.

Iron assay

The iron assay kit obtained from Sigma-Aldrich was used to check the intracellular ferrous iron level in AGS cells. Briefly, cells (2×10^6) were rapidly homogenized in 5 volumes of iron assay buffer on ice and centrifuged at 16,000 × g for 10 min at 4 ℃ to remove insoluble material. The supernatant was collected for measuring ferrous iron according to the manufacturer's instructions.

Quantitative real-time PCR (qRT-PCR)

qRT-PCR was used to evaluate and quantify the mRNA expressions in the present study. Briefly, total cellular RNA was extracted using a Trizol reagent kit (Thermo Fisher Scientific) and quantified on a nanodrop plate using a microplate spectrophotometer (Epoch, BioTek Instruments, Winooski, VT, USA). Equal amounts (500 ng) of total RNA were reverse-transcribed using the AmfiRivert cDNA synthesis kit (GenDEPOT). qRT-PCR was performed using the AmfiSure qGreen Q-PCR master mix (GenDEPOT) following the manufacturer's instructions on the CFX96TM Real-Time RT-PCR System with SYBR®Premix Ex TaqII RT-PCR Kit (TaKaRa Bio Inc., Kusatsu, Japan). All primers were designed and provided by Macrogen (Seoul, Korea). The gene-specific primer sequences used in this study are listed: HO-1 (F, R) 5'-GAATGCTGAGTTCATGAGGAACTT-3', 5'-GCCTTGCGGTGCAGCTCT-3'; GAPDH F (F, R) 5'-ACCACAGTCCATGCCATCAC-3', 5'-CCACCACCCTGTTGCTGTAG-3'. The expression level of target gene was analyzed in triplicates and normalized against the expression level of the endogenous control gene, glyceraldehyde-3-phosphate dehydrogenase (GAPDH).

Western blotting

AGS cells (5 × 10^5 cells) were seeded onto a 60 mm dish (SPL Life Sciences) and stabilized for 24 h. After washing twice with PBS, the cells were treated with samples for 24 h. After rinsing twice with PBS, total protein was isolated from the cell pellets using the Pierce RIPA™ buffer reagent (Thermo Fisher Scientific) containing protease inhibitors (GenDEPOT). A Bio-Rad Protein Assay Kit (Bio-Rad Laboratories Inc., Hercules, CA, USA) was employed to quantify the protein concentration. Equal amounts (50 μg) of total protein

was separated in 10% sodium dodecyl sulfate-polyacrylamide (SDS-PAGE) gel, and the separated proteins were transferred from the gel to a polyvinylidene fluoride (PVDF) membranes (Thermo Fisher Scientific) under a Protein Gel Electrophoresis Chamber System (Thermo Fisher Scientific). The membrane was blocked with PBS-Tween 20 (PBS-T) buffers containing 5% nonfat skim milk at 20-25 ℃ for 2 h and then washed thrice with PBS-T. The membranes were incubated with primary antibodies (dilution, 1:1000) at 4 ℃ overnight. After washing three times with PBS-T, the membranes were incubated with horseradish peroxidase-conjugated secondary antibody (dilution, 1 : 5000) against anti-mouse/rabbit IgG at 20-25 ℃ for 1 h. The membrane was rinsed five times with PBS-T, and the immunoreactive bands were visualized using the West-Q Pico ECL Solution (GenDEPOT) under an Alliance MINI HD9 AUTO Immunoblot Imaging System (UVItec Limited, England, UK). The expression level of each protein blot was quantified using the ImageJ software available in online website (https://imagej.nih.gov/ij/).

Immunofluorescence staining

AGS cells (2×10^5 cells) were grown in 6-well plate (SPL Life Sciences) and treated with samples. After treatment for 24 h, AGS cells were washed with PBS, fixed in cold 4% paraformaldehyde (Samchun Chemical, Seoul, Republic of Korea) for 30 min, and then permeabilizated in 1% Triton X-100 (Samchun Chemical). Cells were further blocked using 5% BSA for 1 h and incubated overnight in primary antibody at 4 ℃. After washing with PBS for three times, cells were followed by 1 h incubation with Alexa Fluor® 488-conjugated goat anti-rabbit IgG (Abcam) in the dark. The fluorescence was observed using a fluorescent microscope (Leica, Wetzlar,

Germany).

***In vivo* xenograft model and experimental schedule**

Male nude mice (CAnN.Cg-Foxn1-nu, 5 weeks) weighing 20-22 g were provided by Orient Bio (Seongnam, South Korea). The mice were housed in a 12 h light-dark cycle chamber, at a temperature of 23 ± 2 °C in a humidity-controlled room (50% humidity) with food and water *ad libitum*. All experimental procedures for animal study were reviewed and approved by the Institutional Animal Care and Use Committee at Kyung Hee University (KHGASP-20-375, approval data: Oct 12th, 2020) and were conducted in accordance with the 2000 Helsinki Declaration. After acclimating the mice for one week, AGS cells (5×10^6) were injected subcutaneously into the right back of the mice. The mice were randomly divided into six groups (n=5) after tumor volume grew to about 100 mm^3: control group, model group, CJ-AuNPs-administered groups with different concentrations (2.5, 5.0, and 10.0 mg/kg), and 5-Fu-administered group. The control and model groups (tumor injection group) received excipient (distilled water), and CJ-AuNPs-administered groups received different doses of CJ-AuNPs, respectively, once daily for 16 days. The 5-Fu-administered group received 5-Fu (5.0 mg/kg) once every 3 days for 16 days as a positive control. All mice were monitored daily for body weight throughout the study period. At the end of administration, all mice were euthanized by CO_2 inhalation at a flow rate of 6 L/min for 3 min, and blood, liver, kidney, and tumor tissues were surgically collected. The liver and kidney were fixed with 10% formalin buffer solution and embedded in paraffin for hematoxylin and eosin (H&E) staining

using H&E solution (Abcam). The tumor tissues were performed by immunohistochemical (IHC) staining using Mouse and Rabbit-specific HRP/DAB (ABC) Detection IHC Kit (Abcam). The stained tissues were visualized using light microscopy (Leica Microsystems, Wetzlar, Germany), and brown-yellow color was used to determine the positive expression of specific proteins in the tumor tissues, which were quantified using ImageJ software.

Table S1

The common compounds in CJ extract

	Analyte Name		Analyte Name
1	Mannose	64	Diphenhydramine N-glucuronide
2	Engeletin	65	Zingerone glucoside
3	Flavanone C-Hex	66	Rutin
4	Galactinol	67	Keracyanin
5	Melibiose	68	Kaempferol-3-O-glucoside-3''-rhamnoside
6	Glucose	69	Astragalin
7	7-hydroxy-2-(4-hydroxyphenyl)-5-[(2S,3R,4S,5S,6R)-3,4,5-trihydroxy-6-(hydroxymethyl)oxan-2-yl]oxy-2,3-dihydrochromen-4-one	70	3,5-Dicaffeoylquinic acids
8	2',3',5'-Triacetyl-5-azacytidine	71	apigetrin
9	Flavone base + 3O, O-AcetylHex	72	4,4'-Difluorobenzhydrol
10	Dictyoquinazol C	73	5,7-dihydroxy-6-methoxy-2-[4-[(2S,3R,4S,5S,6R)-3,4,5-trihydroxy-6-(hydroxymethyl) oxan-2-yl]oxyphenyl]chromen-4-one
11	Adrenochrome o-semiquinone	74	Peonidin-3-O-beta-galactoside
12	Palatinose	75	Citbrasine
13	PTH-(epsilon-phenylthiocarbamyl)lysine	76	Flavone base + 3O, O-HexA, C-Hex, C-Hex
14	Cefadroxil	77	Penicillin V
15	Cordycepin	78	Blepharin
16	Ornidazole	79	12,13-Dihydro-2,10-dihydroxy-6-N-(1-hydroxymethyl-2-hydroxyethylamino)-13-(.beta.-d-glucopyranosyl)-5H-indolo[2,3-a]pyrrolo[3,4-c]carbazole-5,7(6H)-dione
17	Dosulepin	80	Monomethyl tetrachloroterephthalate

18	Gentiobiose	81	Sanguinarine
19	Pizotifen	82	cirsimaritin
20	Apocodeine hydrochloride	83	Cirsimarin
21	Piplartine	84	Aspartyl-Glutamate
22	N-methylasimilobine	85	Malvidin 3-O-galactoside cation
23	Albendazole	86	Khelmarin D
24	Quinate	87	Palmitoleoyl 3-carbacyclic phosphatidic acid
25	1,3-DI(2-METHOXYPHENYL)BENZO(F)QUINAZOLINE	88	Apigenin
26	Trehalose	89	Benzyldiphenylphosphine oxide
27	Lactulose	90	Naphtho[2,3-c]furan-1(3H)-one, 8-(beta-D-glucopyranosyloxy)-9-hydroxy-
28	Maltose	91	Val-Cys-Arg
29	Sucrose	92	Hispidulin
30	Nantenine	93	Aurantio-obtusin
31	N-methyl-2,4-Dihydroxy-3-phenylquinoline"	94	8-[3,5-dihydroxy-6-(hydroxymethyl)-4-(3,4,5-trihydroxyoxan-2-yl)oxyoxan-2-yl]-5,7-dihydroxy-3-(4-hydroxyphenyl)chromen-4-one
32	Eseroline Fumarate	95	N-Undecanoylglycine
33	(E)-8-(4-hydroxy-6-methoxy-7-methyl-3-oxo-1H-2-benzofuran-5-yl)-2,6-dimethyloct-6-enoic acid	96	Dihydro-2,4,6-tris(2-methylpropyl)-4h-1,3,5-dithiazine
34	Norclozapine	97	Muricatacin
35	2-MethylGlutarate	98	Batatasin III
36	16-Phenyltetranorprostaglandin E2	99	4-Hydroxy-3-(3-methyl-2-butenyl)acetophenone
37	Nobiletin	100	(3S)-8-hydroxy-3-methyl-3,4-dihydro-2H-benzo[a]anthracene-1,7,12-trione
38	Hexamethylquercetagetin	101	carboxylic acid
39	Rosmarinic acid	102	Eremopetasinorol

40	2,4-O-Ethylidene-D-erythrose	103	Gingerol
41	Cytidine	104	Vitispirane
42	Sarafloxacin HCl	105	Undecylcarnitine
43	Fluazifop-P-butyl	106	Lys-Thr-Lys
44	Phenol, 2-(2-benzothiazolyl)-	107	3-(2-Furanylmethylene)pyrrolidine
45	Glucose 6-phosphate	108	6-Gingerol
46	Cyclic GMP	109	1-(Benzo[d][1,3]dioxol-4-yl)-2-(ethylamino)propan-1-one
47	Omeprazole	110	Pipercyclobutanamide B
48	L-Glu	111	Phosphatidylcholine lyso 18:2
49	L-5-Oxoproline	112	Dinoterb
50	Duloxetine	113	9,12,15-Octadecatrienoic acid
51	.alpha.-L-Glu-L-Asp	114	2-Benzylidene-1-heptanol
52	Succinate	115	4,6-Decadiyn-1-ol isovalerate
53	Hypoxanthine	116	Triptophenolide
54	Minocycline	117	Methylphenidate
55	Methionyl-Tryptophan	118	3,5-Di-tert-butyl-4-hydroxybenzoic acid
56	Triamifos	119	Octanoylcarnitine-d3
57	Neochlorogenic acid	120	Dioctyl sulfosuccinate
58	Trp	121	5-hydroxypropafenone
59	Salidroside	122	Myristate
60	Chlorogenate	123	D-Fucose
61	Benazeprilat	124	3-Methylhexanal
62	Carbobenzyloxyglycyl-L-norleucine methyl ester	125	Butyl isopropyl disulfide
63	Pheophorbid	126	Dibutyl disulfide

Figure S1

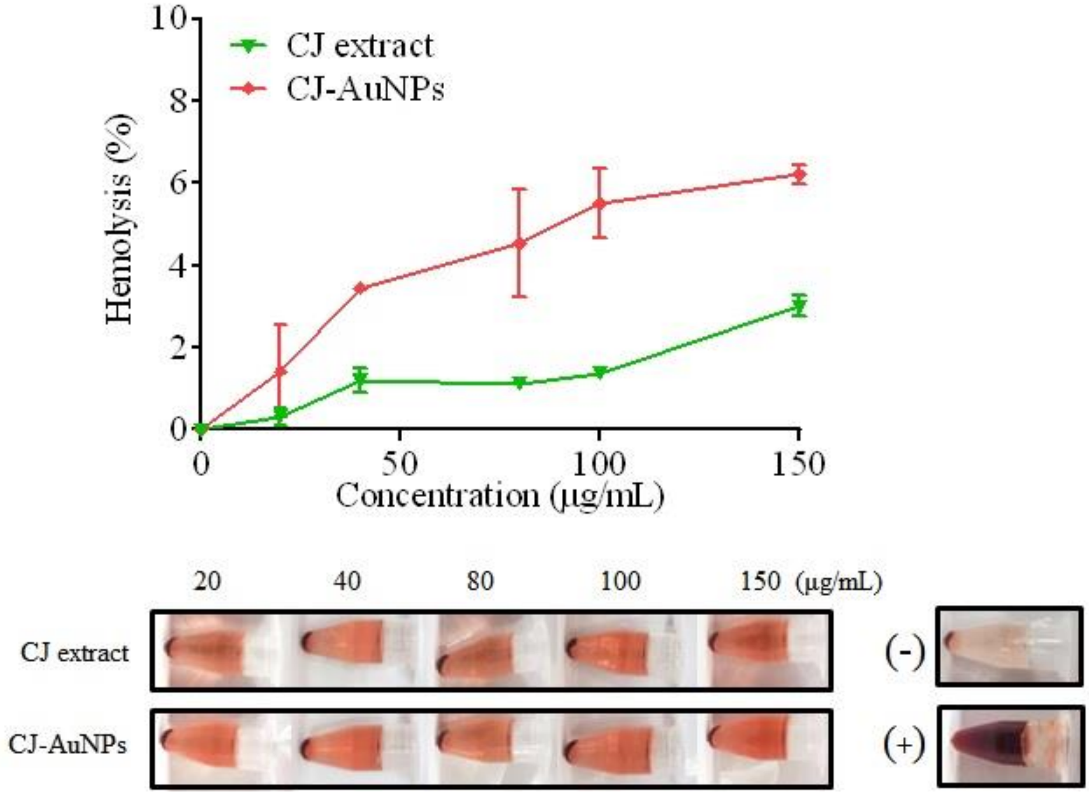

Percentage of hemolysis of red blood cells after exposure to CJ extract and CJ-AuNPs at concentrations of 20.0, 40.0, 80.0, 100.0, and 150.0 μg/mL. Each point represents mean ±S.E., with n = 3

Figure S2

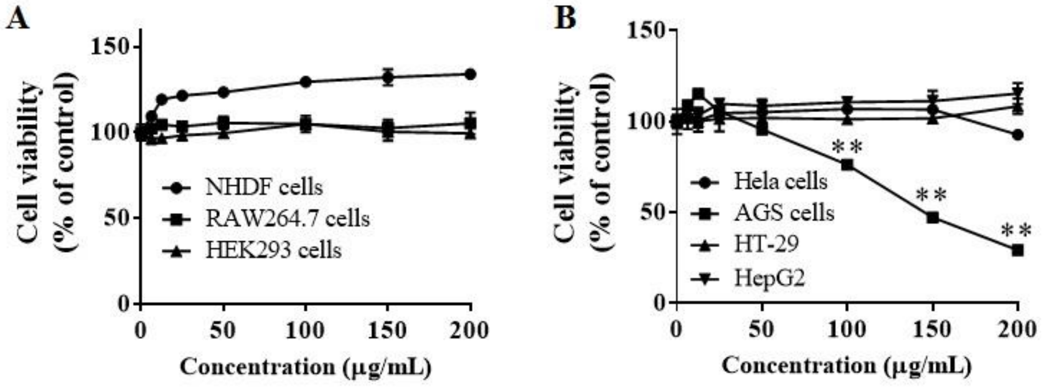

The cytotoxicity of CJ-AuNPs *in vitro*. The effect of CJ-AuNPs on cell viability in normal mammalian cell lines (A) and in cancer cell lines (B)

Figure S3

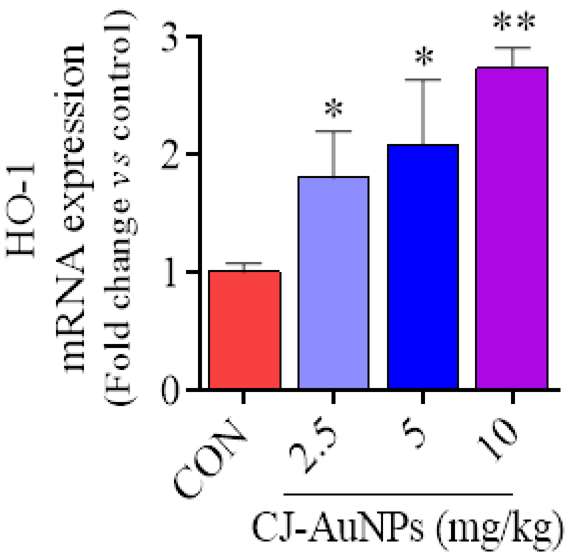

Effect of CJ-AuNPs oral administration on the expression levels of HO-1 mRNAs in tumor tissues isolated from AGS xenograft-bearing mice
